# The neuronal response at extended timescales: a linearized spiking input–output relation

**DOI:** 10.3389/fncom.2014.00029

**Published:** 2014-04-02

**Authors:** Daniel Soudry, Ron Meir

**Affiliations:** Laboratory for Network Biology Research, Department of Electrical EngineeringTechnion, Haifa, Israel

**Keywords:** conductance based neuron models, noise, ion channels, adaptation, power spectral density, linear response, system identification, analytical methods

## Abstract

Many biological systems are modulated by unknown slow processes. This can severely hinder analysis – especially in excitable neurons, which are highly non-linear and stochastic systems. We show the analysis simplifies considerably if the input matches the sparse “spiky” nature of the output. In this case, a linearized spiking Input–Output (I/O) relation can be derived semi-analytically, relating input spike trains to output spikes based on known biophysical properties. Using this I/O relation we obtain closed-form expressions for all second order statistics (input – internal state – output correlations and spectra), construct optimal linear estimators for the neuronal response and internal state and perform parameter identification. These results are guaranteed to hold, for a general stochastic biophysical neuron model, with only a few assumptions (mainly, timescale separation). We numerically test the resulting expressions for various models, and show that they hold well, even in cases where our assumptions fail to hold. In a companion paper we demonstrate how this approach enables us to fit a biophysical neuron model so it reproduces experimentally observed temporal firing statistics on days-long experiments.

## 1. Introduction

Neurons are modeled biophysically using Conductance-Based Models (CBMs). In CBMs, the membrane time constant and the timescales of fast channel kinetics determine the timescale of Action Potential (AP) generation in the neuron. These are typically around 1–20 ms. However, there are various modulating processes that affect the response on slower timescales. Many types of ion channels exist, and some change with a timescale as slow as 10 s (Channelpedia), and possibly even minutes (Toib et al., 1998). Additional new sub-cellular kinetic processes are being discovered at an explosive rate (Bean, 2007; Sjöström et al., 2008; Debanne et al., 2011). This variety is particularly large for very slow processes (Marom, 2010).

For example, ion channels are known to be regulated over the course of long timescales (Levitan, 1994; Staub et al., 1997; Jugloff, 2000; Monjaraz et al., 2000), which could cause changes in ion channel numbers, conductances and kinetics. Also, the ionic concentrations in the cell depend on the activity of the ionic pumps, which can be affected by the metabolism of the network (Silver et al., 1997; Kasischke et al., 2004). Finally, the cellular neurites (De Paola et al., 2006; Nishiyama et al., 2007) and even the spike initiation region (Grubb and Burrone, 2010) can shift their location with time. All these changes can have a large effect on excitability.

Therefore, current CBMs can be considered as strictly accurate only below a certain timescale, since they do not incorporate most of these slow processes. A main reason for this “neglect” is that such slow processes are not well characterized. This is especially problematic since neurons are excitable, so their dynamics is far from equilibrium, highly non-linear and contain feedback. Due to the large number of processes which are unknown or lacking known parameters, it would be hard to simulate or analyze such models. Therefore, it may be hard to quantitatively predict how adding and tuning slow processes in the model would affect the dynamics at longer timescales.

**Figure 1:**
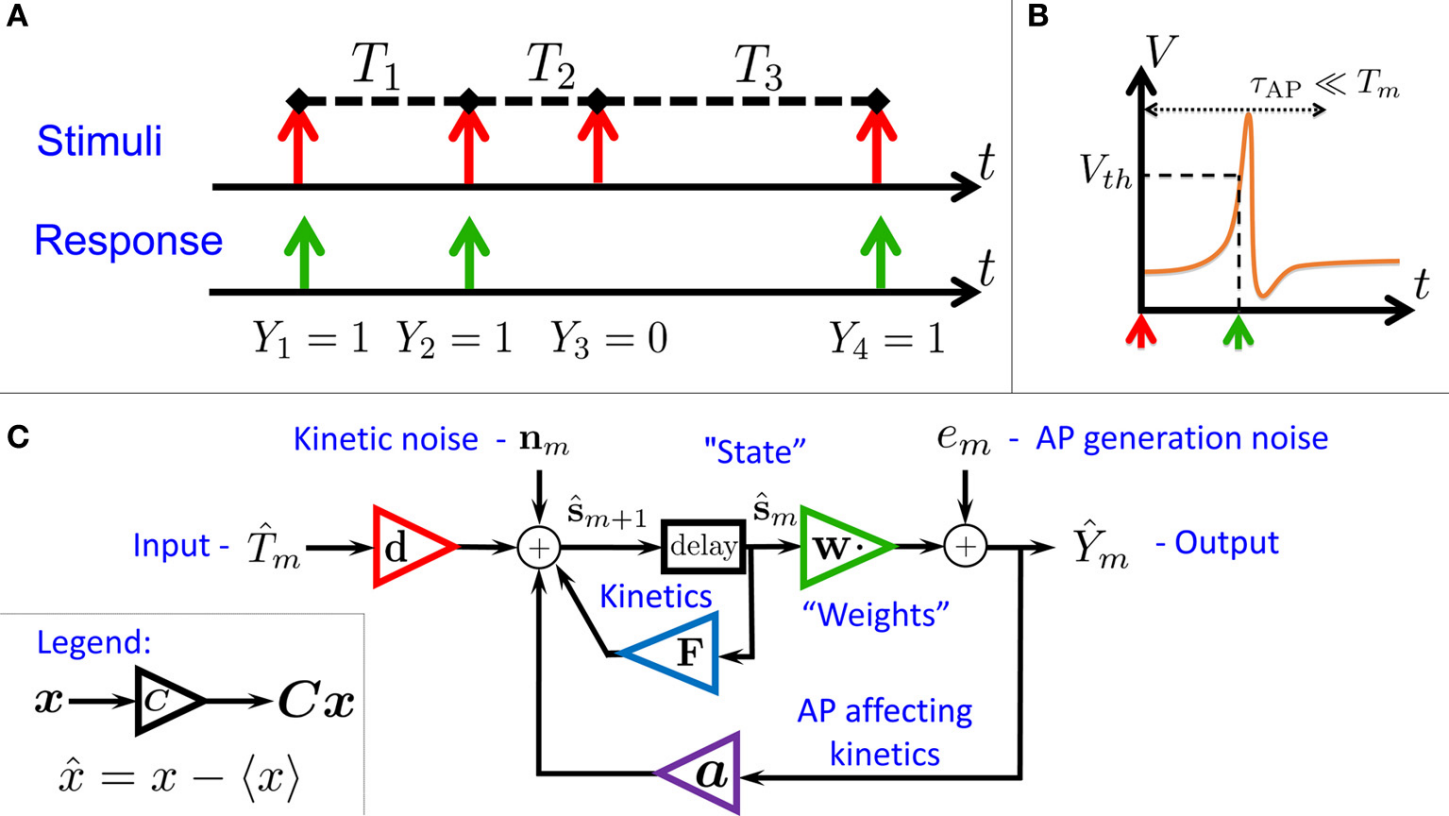
**Schematic summary. (A)** Aim: find the I/O relation between inter-stimulus intervals (*T*_*m*_) and Action Potential (AP) occurrences (*Y*_*m*_) – for a *general* biophysical neuron model (Equations 1–3). **(B)** An AP “occurred” if the voltage *V* crossed a threshold *V*_th_ following the (sparse) stimulus, with *T*_*m*_ ≫ τ_AP_. **(C)** Result: Biophysical neuron model reduced to a simple linear system with feedback (Equations 11, 12), and biophysically meaningful parameters (**F,d,a** and **w**).

In order to allow CBMs with many slow process to be fitted and analyzed, it is desirable to have general expressions that describe their Input–Output (I/O) relation explicitly, based on biophysical parameters. In a previous paper (Soudry and Meir, 2012b), we found that this becomes possible if we use (experimentally relevant Elul and Adey, 1966; Kaplan et al., 1996; De Col et al., 2008; Gal et al., 2010; Goldwyn et al., 2012) sparse spike inputs, similar to the typical output of the neuron (Figures 1A,B). In this case, we derived semi-analytically^1^ a discrete piecewise linear map describing the neuronal dynamics between stimulation spikes, for a general *deterministic* neuron model with a few assumptions (mainly, a timescale separation assumption). Based on this reduced map, we were able to derive expressions for the “mean” behavior of the neuron (e.g., firing modes, firing rate and mean latency).

In this paper, we find that stronger and more general analytical results can be obtained if we take into account the stochasticity of the neuron – arising from ion channel noise^2^ (Neher and Sakmann, 1976; Hille, 2001). Due to the presence of this noise, the discrete map describing the neuronal dynamics is “smoothed out,” and can be linearized. This linearized map constitutes a concise description for the neuronal I/O (Equations 11, 12) based on biophysically meaningful parameters. This I/O is well described by an “engineering-style” block diagram with feedback (Figure 1C), where the input is the process of stimulation intervals and the output is the AP response (Figure 1A). Note that the response is affected both by internal noise and by the input. Beyond conceptual lucidity, such a linear I/O allows the utilization of well known statistical tools to derive all second order statistics, to construct linear optimal estimators and to perform parameter identification. These results hold numerically (Figure 3), even sometimes when our assumptions break down (Figure 4).

In our previous paper, Soudry and Meir (2012b), we used our results to model recent experiments (Gal et al., 2010) where synaptically isolated individual neurons, from rat cortical culture, were stimulated with extra-cellular sparse current pulses for an unprecedented duration of days. Our results enabled us to explain the “mean” response of these neurons. However, the second order-statistics in the experiment seem particularly puzzling. The neurons exhibited 1/*f*^α^ statistics (Keshner, 1982), responding in a complex and irregular manner from seconds to days. In a companion paper (Soudry and Meir, 2014), we demonstrate the utility of our new results. These results allow us to reproduce and analyze the origins of this 1/*f*^α^ on very long timescales.

## 2. Results

This section described our main results in outline. The details of each sub-section here appear in the corresponding sub-section in section 4. For readers who do not wish to go through the detailed derivations, the present section is self-contained. Readers who do wish to follow the mathematical derivations, should first read section 4, where, for convenience, each subsection (except for the last one) can be read independently. In our notation 〈·〉 is an ensemble average, $i≜\sqrt{-1}$, a non-capital boldfaced letter **x** ≜ (*x*_1_, …, *x*_*n*_)^⊤^ is a column vector (where (·)^⊤^ denotes transpose), and a boldfaced capital letter **X** is a matrix (with components *X*_*mn*_).

### 2.1. Full model

The voltage dynamics of an isopotential neuron are determined by ion channels, protein pores which change their conformations stochastically with voltage-dependent rates (Hille, 2001). At the population level, such dynamics are generically described by Fox and Lu (1994), Goldwyn et al. (2011), and Soudry and Meir (2012b) a CBM

(1) $$\dot{V}=f\left(V,r,s,I\left(t\right)\right)$$

(2) $$\dot{r}=A_{r}\left(V\right)r+B_{r}\left(V,r\right)\xi_{r}$$

(3) $$\dot{s}=A_{s}\left(V\right)s+B_{s}\left(V,s\right)\xi_{s},$$

with **voltage** *V*, stimulation current *I*(*t*), **rapid** variables **r** (e.g., *m, n, h* in the Hodgkin–Huxley (HH) model Hodgkin and Huxley, 1952), **slow** “excitability” variables **s** (e.g., slow sodium inactivation Chandler and Meves, 1970), rate matrices **A**_*r/s*_, white noise processes ξ_*r/s*_ (with zero mean and unit variance), and matrices **B**_*r/s*_ which can be written explicitly using the rates and ion channel numbers (Orio and Soudry, 2012) (**D** = **BB**^⊤^ is the diffusion matrix Orio and Soudry, 2012). For simplicity, we assumed that **r** and **s** are not coupled directly, but this is non-essential (Contou-Carrere, 2011; Wainrib et al., 2011). The parameter space can be constrained (Soudry and Meir, 2012b), since we consider here only *excitable*, non-oscillatory neurons which do not fire spontaneously^3^ and which have a single resting state – as is common for cortical cells, e.g., Gal et al. (2010).

Since the components of **r** and **s** usually represent fractions, in some cases it is more convenient to use the normalization constraint (i.e., that fractions sum to one), and reduce the dimensions of **r, s**, and ξ_*r/s*_. After this reduction, the form of Equations (1–3) changes to

(4) $$\dot{V}=f\left(V,r,s,I\left(t\right)\right)$$

(5) $$\dot{r}=A_{r}\left(V\right)r-b_{r}\left(V\right)+B_{r}\left(V,r\right)\xi_{r},$$

(6) $$\dot{s}=A_{s}\left(V\right)s-b_{s}\left(V\right)+B_{s}\left(V,s\right)\xi_{s},$$

where all the variables and parameters have been redefined (with their size decreased). Note that we have slightly abused notation by using the same symbols in Equations (4–6) and in Equations (1–3). The specific set of equations used will always be stated. We call Equations (4–6) the “compressed form” of the CBM.

Such biophysical neuronal models (either Equations 1–3 or 4–6) are generally complex and non-linear, containing many variables and unknown parameters (sometimes ranging in the hundreds Koch and Segev, 1989; Roth and Häusser, 2001), not all of which can be identified (Huys et al., 2006). Therefore, such models are notoriously difficult to tune, highly susceptible to over-fitting and computationally expensive (Migliore et al., 2006; Gerstner and Naud, 2009; Druckmann et al., 2011). Also, the high degree of non-linearity usually prevents exact mathematical analysis of such models at their full level of complexity (Ermentrout and Terman, 2010). However, much of the complexity in such models can be overcome under well defined and experimentally relevant settings (Elul and Adey, 1966; Kaplan et al., 1996; De Col et al., 2008; Gal et al., 2010; Goldwyn et al., 2012), if we use sparse inputs, similar in nature to the spikes commonly produced by the neuron.

### 2.2. Model reduction

We consider a stimulation setting motivated by the experiments described in Gal et al. (2010) and further elaborated on in section 3. Specifically, suppose *I*(*t*) consists of a train of pulses arriving at times {*t*_*m*_} (Figure 1A, *top*), so *T*_*m*_ = *t*_*m* + 1_ − *t*_*m*_ ≫ τ_AP_ with τ_AP_ being the timescale of an AP (Figure 1B). Our aim is to describe the AP occurrences *Y*_*m*_, where *Y*_*m*_ = 1 if an AP occurred immediately after the *m*-th stimulation, and 0 otherwise (Figure 1A, *bottom*). Recall again that we assume the neuron does not generate APs unless stimulated (as observed in Gal et al., 2010).

In this section we “average out” Equations (1–3) using a semi-analytical method similar to that in Soudry and Meir (2012b). To do so, we need to integrate Equations (1–3) between *t*_*m*_ and *t*_*m* + 1_. Since *T*_*m*_ ≫ τ_AP_, the rapid AP generation dynamics of (*V*, **r**) relax to a steady state before *t*_*m* + 1_. Therefore, the neuron AP “remembers” any history before *t*_*m*_ only through **s**_*m*_ = **s**(*t*_*m*_). Given **s**_*m*_, the response of the fast variables (*V*, **r**) to the *m*-th stimulation spike will determine the probability to generate an AP. This probability,

$$p_{\text{AP}}\left(s_{m}\right)≜P\left(Y_{m}|s_{m}\right)=\langle Y_{m}|s_{m}\rangle ,$$

collapses all the relevant information from Equations (1, 2), and can be found numerically from the pulse response of Equations (1, 2) with **s** held fixed (section 4.2.4).

In order to integrate the remaining Equation (3), we define *X*_+_, *X*_−_ and *X*_0_ to be the averages of a quantity *X*_*s*_ during an AP response, a failed AP response and rest, respectively ^4^. Also, we denote

(7) $$\begin{array}{l} X\left(Y_{m},T_{m}\right)\overset{\Delta }{=}\tau_{\text{AP}}T_{m}^{-1}\left(Y_{m}X_{+}+\left(1-Y_{m}\right)X_{-}\right)+\\ \;\left(1-\tau_{\text{AP}}T_{m}^{-1}\right)X_{0}, \end{array}$$

as the steady state mean value of *X*_*s*_. For analytical simplicity we assume^5^ *T*_*m*_ ≪ τ**_s_**. We obtain, to first order

(8) $$s_{m+1}=s_{m}+T_{m}A\left(Y_{m},T_{m}\right)s_{m}+n_{m}.$$

where **n**_*m*_ is a white noise process with zero mean and variance *T*_*m*_**D** (*Y*_*m*_, *T*_*m*_). For the compressed form (Equations 4–6) we have instead

(9) $$s_{m+1}=s_{m}+T_{m}\left[A\left(Y_{m},T_{m}\right)s_{m}-b\left(Y_{m},T_{m}\right)\right]+n_{m}.$$

Note that such a simplified discrete time map, which describes the excitability dynamics of the neuron, has far fewer parameters than the full model, since it is written explicitly only using the averaged microscopic rates of **s** (through **A** and **D**), population sizes (through **D**), the probability to generate an AP given **s**, *p*_AP_ (**s**), and the relevant timescales. This effective model exposes the large degeneracy in the parameters of the full model and leads to significantly reduced simulation times and mathematical tractability. Notably, the dynamics of the state **s**_*m*_ (Equation 8) depends on the input *T_m_ and* the output *Y*_*m*_ – and this feedback affects all of our following results.

### 2.3. Linearization

**Figure 2:**
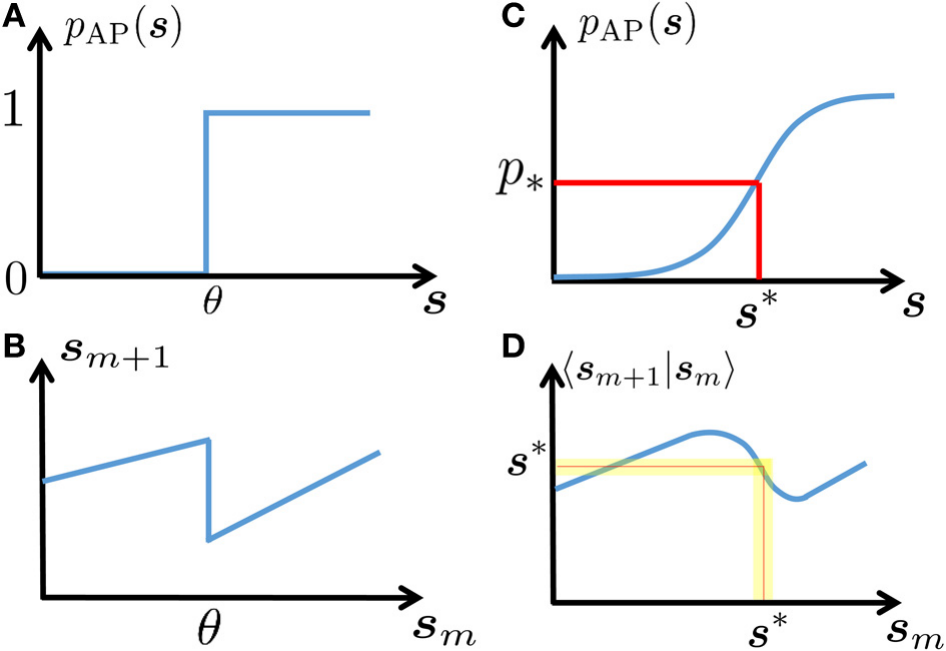
**Schematic explanation of linearization**. In a deterministic neuron, an AP will be generated in response to stimulation if and only if the neuronal excitability (here, **s**) is above a certain threshold **(A)**. This generates discontinuous dynamics in the neuronal excitability **(B)**, see Equations 7, 8). In a stochastic neuron, the response probability is a smooth function of **s (C)**. In turn, this “smooths” the dynamics **(D)**. Note that if the noise is sufficiently high (as is true in many cases, for biophysically realistic levels of noise), then this generates a stable fixed point s^*^ – which gives the mean response probability p_*_, and around which the dynamics can be linearized (yellow region).

In this section we exploit the intrinsic ion channel noise to linearize the neuronal dynamics, rendering it more tractable than the (less realistic) noiseless case (Soudry and Meir, 2012b). Suppose that the inter-stimulus intervals {*T*_*m*_} have stationary statistics with mean T_*_ so that τ_AP_ ≪ *T*_*m*_ ≪ τ**_s_** with high probability. Since **s** is slow and AP generation is rather noisy in this regime (Soudry and Meir, 2012b) (so *p*_AP_ (**s**_*m*_) is slowly varying), we assume that a stable excitability fixed point s_*_ exists (Figure 2). Therefore, the perturbations ${\hat{s}}_{m}=s_{m}-s_{*}$ are small and we can linearize

$$p_{\text{AP}}\left(s_{m}\right)\approx p_{*}+w^{⊤}{\hat{s}}_{m}.$$

Denoting X_*_ = *X*(p_*_, T_*_), the mean AP firing rate can be found self consistently from the location of the fixed point s_*_,

(10) $$\langle Y_{m}\rangle =p_{*}=p_{\text{AP}}\left(s_{*}\right),$$

where s_*_ depends on p_*_ through A_*_s_*_ = 0, or s_*_ = **A**^−1^_*_b_*_ in the compressed form.

The perturbations ${\hat{s}}_{m}=s_{m}-s_{*}$ around the fixed point s_*_ are described by the linear system

(11) $${\hat{s}}_{m+1}=F{\hat{s}}_{m}+d{\hat{T}}_{m}+a{\hat{Y}}_{m}+n_{m},$$

(12) $${\hat{Y}}_{m}=w^{⊤}{\hat{s}}_{m}+e_{m},$$

where ${\hat{T}}_{m}=T_{m}-T_{*}$, ${\hat{Y}}_{m}=Y_{m}-\langle Y_{m}\rangle$, **F** ≜ **I** + T_*_A_*_, 〈**n**_*m*_**n**^⊤^_*m*_〉 = T_*_D_*_, *e*_*m*_ is a (*non*-Gaussian) white noise process, 〈*e*_*m*_〉 = 〈*e*_*m*_**n**_*m*_〉 = 0, σ^2^_*e*_ ≜ 〈*e*^2^_*m*_〉 = p_*_ (1 − p_*_), **d** ≜ **A**_0_s_*_ and **a** ≜ τ_AP_ (**A**_+_ − **A**_−_) s_*_. If we use the compressed form instead, then these results remain valid, except we need to re-define **d** ≜ **A**_0_s_*_ − **b**_0_ and **a** ≜ τ_AP_[(**A**_+_ − **A**_−_) s_*_ − (**b**_+_ − **b**_−_)].

The *linear* I/O for the fluctuations in Equations (11, 12), which contains feedback from the “output” ${\hat{Y}}_{m}$ to the state variable ${\hat{s}}_{m}$ (Figure 1C), can be very helpful mathematically and its parameters are directly related to biophysical quantities.

### 2.4. Linear systems analysis

Using standard tools, this formulation makes it now possible to construct optimal linear estimators for *Y*_*m*_ and **s**_*m*_ (Anderson and Moore, 1979), perform parameter identification (Lejung, 1999), and find all second order statistics in the system (Papoulis and Pillai, 1965; Gardiner, 2004), such as correlations or Power Spectral Densities (PSD). For example, for *f* ≪ *T*^−1^_*_, the PSD of the output is

(13) $$\begin{array}{l} S_{Y}\left(f\right)=w^{⊤}H_{c}\left(-f\right)\left(D_{*}+T_{*}^{-2}dd^{⊤}S_{T}\left(f\right)\right)H_{c}^{⊤}\left(f\right)w\\ \;+T_{*}\sigma_{e}^{2}{\left|1+T_{*}^{-1}w^{⊤}H_{c}\left(f\right)a\right|}^{2} \end{array}$$

where

(14) $$H_{c}\left(f\right)\overset{\Delta }{=}{\left(2\pi fi-A_{*}-T_{*}^{-1}aw^{⊤}\right)}^{-1}.$$

Similarly, the PSD of the state variables is

(15) $$S_{s}\left(f\right)=H_{c}\left(-f\right)\left(D_{*}+T_{*}^{-1}aa^{⊤}\sigma_{e}^{2}+T_{*}^{-2}dd^{⊤}S_{T}\left(f\right)\right)H_{c}^{⊤}\left(f\right),$$

and the input–output cross-PSD is

(16) $$S_{YT}\left(f\right)=T_{*}^{-1}w^{⊤}H_{c}\left(-f\right)dS_{T}\left(f\right).$$

Again, note the large degeneracy here – many different sets of parameters will generate the same PSD. Using similar methods, the PSDs of various response features, such as the AP latency or amplitude, can also be derived (Equation 124).

Finally, we note Equations (11) and (12) can be re-arranged as a *direct* I/O relation. First, we define the filters (transfer functions)

(17) $$H^{\text{ext}}\left(f\right)≜T_{*}^{-1}w^{⊤}H_{c}\left(f\right)d$$

(18) $$H^{\text{int}}\left(f\right)≜\left(T_{*}^{-1}w^{⊤}H_{c}\left(f\right)K+1\right)\sigma_{v}$$

where **K** = **a** + **FPw**σ^−2^_*v*_ and σ^2^_*v*_ = **w**^⊤^ **Pw** + σ^2^_*e*_,, with **P** being the solution of

(19) $$P=FPF^{⊤}-{\left(w^{⊤}Pw+\sigma_{e}^{2}\right)}^{-1}FPww^{⊤}PF^{⊤}+T_{*}D_{*}.$$

Using these filters, we obtain, in the frequency domain,

(20) $$\hat{Y}\left(f\right)=H^{\text{ext}}\left(f\right)\hat{T}\left(f\right)+H^{\text{int}}\left(f\right)z\left(f\right),$$

where $\hat{Y}\left(f\right),\hat{T}\left(f\right)$ and *z*(*f*) are the Fourier transforms of *Y*_*m*_, ${\hat{T}}_{m}$ and *z*_*m*_, respectively, with *z*_*m*_ being a white noise process with zero mean and unit variance. Notably, these transfer functions can be identified from the spiking input–output of the neuron $\left\{{\hat{T}}_{m},{\hat{Y}}_{m}\right\}$, without access to the underlying dynamics or biophysical parameters. Specifically, Equation (20) has the form of an ARMAx(*M, M, M*) model^6^ (Lejung, 1999) (recall *M* is the dimension of **s**), which can be estimated using standard tools (e.g., the system identification toolbox in Matlab).

### 2.5. Numerical tests

As we argued so far, a main asset of the present approach is its applicability to a broad range of models of various degrees of complexity and realism. Recall that our three assumptions are

1. τ_AP_ ≪ *T*_*m*_ (temporally sparse input).
2. *T*_*m*_ ≪ τ**_s_** (timescale separation).
3. A stable excitability fixed point s_*_ exists, (“noisy” neuron).

In this section we will demonstrate that our analytical approximations agree very well with the numerical solution of Equations (1–3), even in some cases where the assumptions 2 and 3 do not hold. Therefore, these assumptions are sufficient, but not necessary.

#### 2.5.1. The HHS model

**Figure 3:**
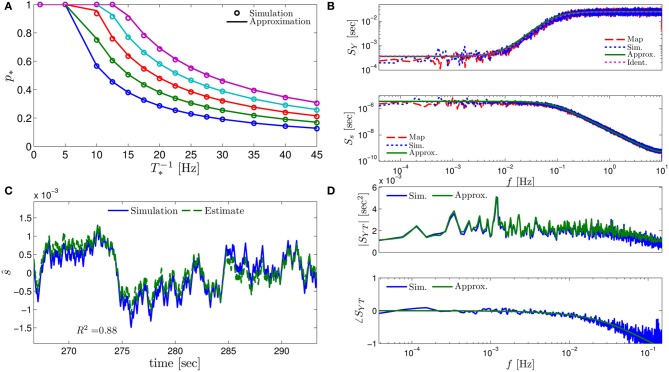
**Comparing the mathematical results with the numerical simulation of the full model (Equations 1–3) for the stochastic HHS model (section 4.5.1)**. **(A)** Firing probability p_*_(*T*^−1^_*_) (Equation 10) for different currents (*I*_stim_ = 7.5, 7.7, 7.9, 8.1, 8.3 μA from bottom to top). **(B)** The PSDs *S*_*Y*_(*f*) and *S*_*s*_(*f*). “Sim” is a simulation of the full model, “Map” is a (10^4^ faster) simulation of Equation (8) together with *p*_AP_ (**s**_*m*_), while “Approx” refers to the analytical expressions (Equations 13–15). “Ident” is the PSD *S*_*Y*_(*f*) of the linear system identified from the spiking data. Note the high/low-pass filter shapes of *S*_*Y*_(*f*) and *S*_*s*_(*f*), respectively. **(C)** Optimal linear estimation of $\hat{s}$. **(D)** Amplitude and phase of the cross-spectrum *S*_*YT*_(*f*) for Poisson stimulation (Equations 16). Note that the frequency range was cut due to spectral estimation noise (see Figure 8). Parameters: *I*_0_ = 7.9 μA and T_*_ = 50 ms in **(B–D)**, and also stimulation is periodical in **(A–C)**. Note the low-pass filter shapes of *S*_*YT*_(*f*).

First, in Figure 3 we tested our results on the HH model with Slow sodium inactivation. This “HHS” model (Soudry and Meir, 2012b, and see section 4.5.1 for parameter values) augments the classic HH model (Hodgkin and Huxley, 1952) with an additional slow inactivation process of the sodium conductance (Chandler and Meves, 1970; Fleidervish et al., 1996). The HHS model includes the uncoupled stochastic Hodgkin–Huxley (HH) model equations (Fox and Lu, 1994), and is written in the compressed formulation (Equations 4–6)

(21) $$\begin{array}{l} C\dot{V}={\bar{g}}_{Na}sm^{3}h\left(E_{Na}-V\right)+{\bar{g}}_{K}n^{4}\left(E_{K}-V\right)\\ \;+\;{\bar{g}}_{L}\left(E_{L}-V\right)+I\left(t\right) \end{array}$$

(22) $$\begin{array}{l} \dot{r}=\left[\alpha_{r}\left(V\right)\left(1-r\right)-\beta_{r}\left(V\right)r\right]\phi +\\ \;\sqrt{N^{-1}\phi \left(\alpha_{r}\left(V\right)\left(1-r\right)+\beta_{r}\left(V\right)r\right)}\xi_{r}, \end{array}$$

for *r* = *m, n* and *h*, with the additional kinetic equation for slow sodium inactivation

(23) $$\dot{s}=\delta \left(V\right)\left(1-s\right)-\gamma \left(V\right)s+\sqrt{N^{-1}\left(\delta \left(V\right)\left(1-s\right)+\gamma \left(V\right)s\right)}\xi_{s},$$

where *V* is the membrane voltage, *I*(*t*) is the input current, *m, n* and *h* are ion channel “gating variables,” α_*r*_(*V*), β_*r*_(*V*), δ(*V*), and γ(*V*) are the voltage dependent kinetic rates of these gating variables, ϕ is an auxiliary dimensionless number, *C* is the membrane's capacitance, *E*_*K*_, *E*_*Na*_ and *E*_*L*_ are ionic reversal potentials, *g*_*K*_, *g*_*Na*_ and *g*_*L*_ are ionic conductances and *N* is the number of ion channels. Note that in this model τ_*s*_ is between 20 s (at rest) and 40 s (during an AP).

In Figure 3A we show that through Equation (10) we can accurately calculate p_*_, the mean probability to generate an AP (so p_*_T^−1^_*_ is the firing rate of the neuron). In Figure 3B we demonstrate both the analytical expression (Equations 13, 15), or a simulation of the reduced model (Equation 8), will give the PSDs *S*_*Y*_ (*f*) or *S*_*s*_ (*f*) of the full model (Equations 1–3). In Figure 3D we do the same for the analytical expression (Equation 16) of the Cross-PSD *S*_*YT*_ (*f*). In Figure 3C we show that we can construct a linear optimal filter for the internal state ${\hat{s}}_{m}$, given $\left\{{\left\{T_{k}\right\}}_{k\;=\;0}^{m\;-\;1},{\left\{Y_{k}\right\}}_{k\;=\;0}^{m\;-\;1}\right\}$ quite well, with low mean square error (section 4.4.4). Finally, back in Figure 3B, *top*, we infer the linear model parameters from the spike output using system identification tools [here, with ARMAx(1, 1, 1)], and present the PSD of the identified model (“Ident”). Since *S*_*Y*_ (*f*) = |*H*_int_ (*f*)|^2^ (see Equation 111) for periodical input (in which ${\hat{T}}_{m}=0$) this allows us to confirm that the linear model was identified. As can be seen, the identified filter matches well with that of the linear system.

#### 2.5.2. Testing the limit of our assumptions

**Figure 4:**
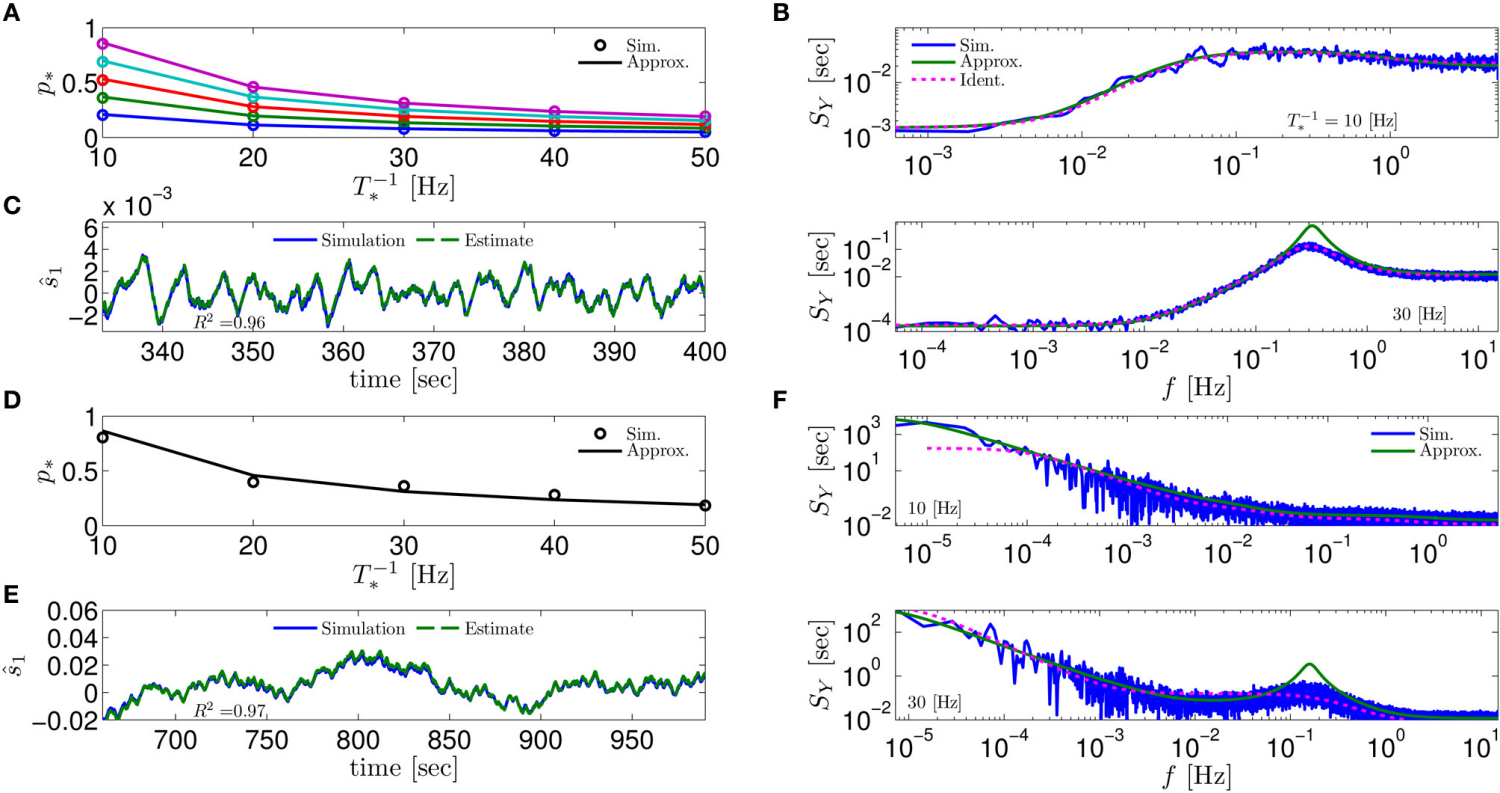
**Comparing mathematical results with full model simulation when the assumptions fail to hold**. In the HHSIP model (HHS with potassium inactivation) we plot **(A)** p_*_(*T*^−1^_*_) for different currents (*I*_0_ = 7.5, 7.7, 7.9, 8.1, 8.3μA from bottom to top). **(B)** *S*_*Y*_(*f*) for two values of T_*_. As before, “Sim” is a simulation of the full model, “Approx” is the analytical approximation, and “Ident” is the PSD *S*_*Y*_(*f*) of the linear system identified from the spiking data. Upper figure shows the case when T_*_ ≈ 0.5τ**_s_** so the timescale separation assumption breaks down. In the lower figure the parameters are close to a Hopf bifurcation where a limit cycle is formed so the fixed point assumption breaks down, so the estimation of the limit cycle frequency component is less accurate. **(C)** The estimation of ${\hat{s}}_{1}$ for *T*^−1^_*_ ≈ 30 Hz is even better than in the HHS case. Similarly to **(A–C)** we plot the results of the HHMSIP model (HHSIP with many additional slow sodium inactivation kinetics) in **(D–F)**, which has considerably more noise in the slow kinetics, and so even larger fluctuations (which further invalidates the fixed point assumption). See section 4.5 for various model details.

**Figure 5:**
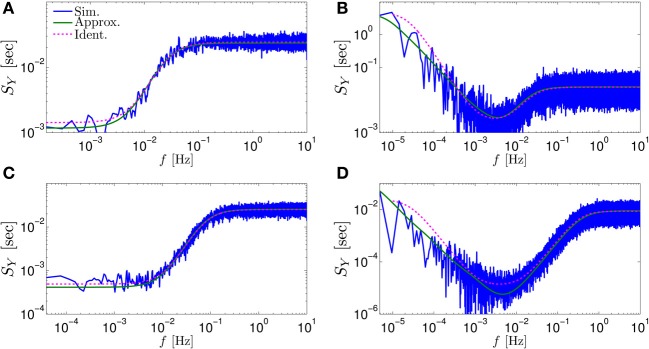
**Comparing mathematical results (green) with full model simulation (blue) for various models. (A)** Coupled HHS (HHS coupled slow and rapid kinetics) **(B)** HHMS (HHS with many additional slow sodium inactivation kinetics) **(C)** HHSTM (HHS with a synapse) **(D)** Multiplicative HHMS (variant of HHMS). As before, “Sim” is a simulation of the full model, “Approx” is the analytical approximation, and “Ident” is the PSD *S*_*Y*_(*f*) of the linear system identified from the spiking data. See section 4.5 for various model details.

Next, we demonstrate that our analytical expressions hold also for various other models. Specifically, in the following scenarios: (1) when the kinetics of the neuron are extended to arbitrarily slow timescales, (2) when the assumptions 2 and 3 break down, (3) when the rapid and slow kinetics are coupled, (4) when “physiological” synaptic inputs are used. These results are presented in Figures 4, 5, with specific model parameters given in section 4.5.

First, we tested whether or not the model can be extended to arbitrarily slow timescales. We added to the HHS model four types of slow sodium inactivation processes with increasingly slower kinetics and smaller channel numbers. In the first case, those processes were added additively (as different currents), so *s* was replaced with ∑_*i*_ *s*_*i*_ in the voltage equation (Equation 21). This model was denoted “HHMS” (HH with Many Sodium slow inactivation processes, section 4.5.4). In the second case, those processes were added in a multiplicative manner (as different processes affecting the same channel, in the uncoupled approximation), so *s* was replaced with ∏_*i*_ *s*_*i*_ in the voltage equation (Equation 21). We denote this model as “Multiplicative HHMS” (section 4.5.5). In both cases, our analytical approximations seemed to hold quite well. For example, the approximated *S*_*Y*_ (*f*) (Equation 13) corresponded rather well with the numerical simulation of the full model (Figures 5B,D, respectively).

Next, to test the limits of our assumptions we extended the HHS model to the HHSIP model (from Soudry and Meir, 2012b, see section 4.5.6) and added a potassium inactivation current which had faster kinetics (so τ**_s_** ≈ 5 Hz). So if *T*^−1^_*_ = 10 Hz, we get T_*_ ≈ 0.5τ**_s_**, so the timescale separation assumption 2 is not strictly valid here. Also, for certain parameter values we get a limit cycle in the dynamics of ${\hat{s}}_{m}$, so the fixed point assumption 3 fails. However, it seems that our approximations still follow the numerical simulation of the full model: for p_*_ at various stimulation frequencies *T*^−1^_*_ and currents *I*_0_ (Figure 4A), for *S*_*Y*_ (*f*) at *T*^−1^_*_ = 10 Hz when assumption 2 breaks down (Figure 4B, *top*), for *S*_*Y*_ (*f*) at *T*^−1^_*_ = 30 Hz when assumption 3 breaks down (near a Hopf bifurcation) and a limit cycle begins to form (see Figure 4B, *bottom*), and for state estimation of ${\hat{s}}_{1}$ using a linear optimal filter, again at *T*^−1^_*_ = 10 Hz (Figure 4C).

The only discrepancy seemed to appear in the limit cycle case, where the frequency of the limit cycle “sharpens” the peak in *S*_*Y*_ (*f*) (Figure 4B, *bottom*). This may suggest that, in this case, the perturbations of the system near the limit cycle could be linearized, and that the eigenvalues of that linearized system might be related to the eigenvalues of the linearized system around the (now unstable) fixed point s_*_. More generally, the results so far indicate that even if our assumptions are inaccurate, it is possible that the resulting error will not accumulate and remain small – in comparison with the intrinsic noise in the model.

**Figure 6:**
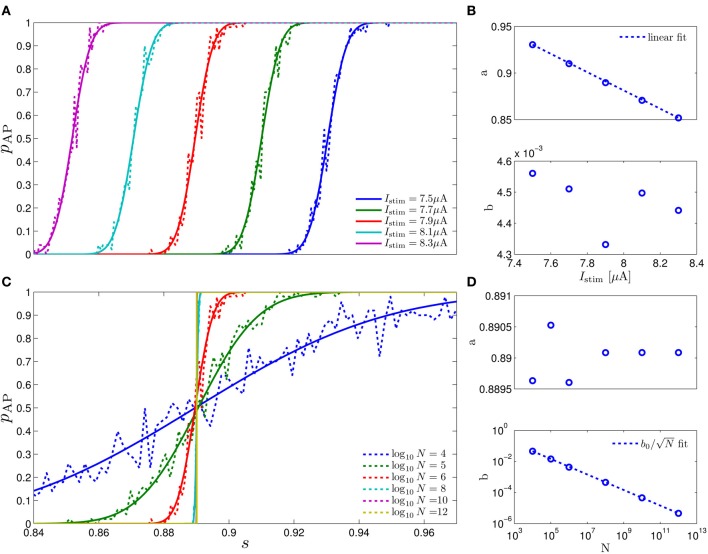
**Fitting of ***p*_AP_**(***s***) **=** Φ((***s*** − ***a***)/***b***) in the HHS model. (A)** Fitting of *p*_AP_ (*s*) for various values of *I*_0_. **(B)** Fitting shows that *a* is linearly decreasing in *I*_0_. **(C)** Fitting of *p*_AP_ (*s*) for various values of *N*. **(D)** Fitting shows that $b\propto 1/\sqrt{N}$.

Next, to challenge the approximation even more, we added to the HHSIP model four types of sodium currents with increasingly slower kinetics and fewer channels, similarly to the HHMS model (so this is the “HHMSIP” model, section 4.5.7). This significantly increased the variance of the dynamic noise **n**_*m*_, rendering the dynamics more “noisy.” These random fluctuations in **s**_*m*_ (Figure 4E) are of similar magnitude to the width of the threshold (non-saturated) region in *p*_AP_ (**s**_*m*_) (see Figure 6). This renders the fixed point assumption 3 inaccurate, since now the linear approximation $p_{\text{AP}}\left(s_{m}\right)\approx p_{*}+w^{⊤}{\hat{s}}_{m}$ breaks down most of the time. However, even in this case, the approximations seem to hold quite well with simulations of the full neuronal model (Figures 4D–F).

In Figure 5A we used a coupled version of the HHS model (“coupled HHS” model, section 4.5.2), in which the equations for **r** and **s** in the full model are tangled together, and not separated as we assumed in Equations (2, 3). Even in this case, our approximations seemed to hold well.

Finally, in Figure 5C, we extend the HHS model so that the stimulations are not given directly, but through a synapse. We used the biophysical Tsodyks–Markram model (Tsodyks and Markram, 1997) of a synapse with short-term depression, with added stochasticity (“HHSTM” model, section 4.5.3). This also seemed to work well.

In all simulation we also added the PSD of the linear model identified from the spike output (“Ident.”), to show that it can be estimated reasonably well. Note that the performance at the lowest frequencies seems to be significantly worse when they contain relatively high power. This is not surprising since it is typically harder to estimate model parameters, when the data has such (1/*f*^α^) PSD shape – which indicates long-term correlations (Beran, 1992).

## 3. Discussion

**Figure 7:**
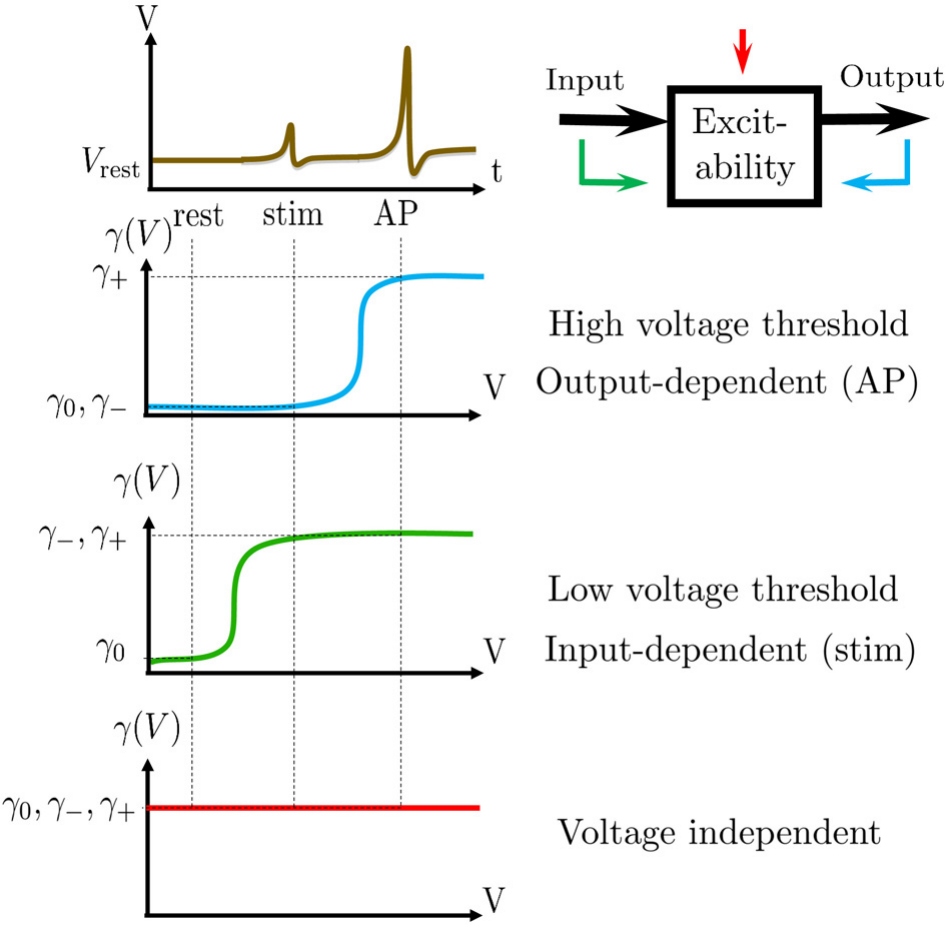
**The averaged kinetic rates**. **Left**: The averaged rates demonstrated for three common kinetic rates γ(*V*) with sigmoidal shapes. **Right**: The voltage threshold of the sigmoid determines whether the process is sensitive to APs (the output), stimulation pulse (the input), or neither. Note that a similar classification of biophysical processes affecting excitability was previously suggested inWallach (2012, Figure 3.1).

In this work we found that under a temporally sparse (“spike-like”) stimulation regime (Figures 1A,B) we can perform accurate semi-analytical linearization of the spiking input–output relation of a CBM (Figure 1C), while retaining biophysical interpretability of the parameters (e.g., Figure 7). This linearization considerably reduces model complexity and parameter degeneracy, and enables the use of standard analysis and estimation tools. Importantly, this method is rather general, since it can be applied to any stochastic CBM, with only a few assumptions.

### 3.1. Connection to previous work

To the best of our knowledge, such results are novel, as no previous work examined analytically the response of general stochastic CBMs to temporally sparse input for extended durations. However, the connection between sparse inputs and slow timescales has been previously made. It was previously suggested (Linaro et al., 2011) that sparse inputs could be used to identify neuronal parameters in a network of integrate and fire neurons with spike frequency adaptation. Interestingly, using different methods we reach a qualitatively similar conclusion here, though not in a network setting, and for a different class of neuron models.

Additionally, in Soudry and Meir (2012b) we modeled neurons under *periodical* stimulation using *deterministic* CBMs with slow kinetics, which are completely *uncoupled* from each other, and slower than the stimulation rate. Using a reduction scheme similar in nature to that described here, we were able to describe the deterministic CBM's excitability and response using a discrete-time map – which “samples” the neuronal state at each stimulation. Analyzing this map, we obtained analytical results describing the neuronal activation modes, spike latency dynamics, mean firing rate and short-time firing patterns. Stochastic CBMs were then examined numerically, and were shown to lead to qualitatively different responses, which are more similar to the experimentally observed responses.

The current work, therefore, generalizes this previous work. Here, we analyze the general case of *stochastic* CBMs, under *general* sparse stimulation patterns and with *coupled* slow kinetic dynamics. Therefore, the framework in the previous work (Soudry and Meir, 2012b) could be considered as a special case of this work, in which there is an infinite number of ion channels (*N* → ∞, so **B**_*r/s*_ = **D**_*r/s*_ = 0), *T*_*m*_ = T_*_ (so ${\hat{T}}_{m}=0$) and **A**_*s*_ (*V*) (the rate matrix) is a diagonal matrix. In the current work we similarly show that, in the generalized framework, the CBM's excitability and responses can be succinctly described using a discrete-time map. It is then straightforward to derive results paralleling those in Soudry and Meir (2012b) in this more general setting, such as the mean firing rate (Equation 10).

### 3.2. Theoretical novelty

However, the main novelty lies in our additional results, that could not be derived in Soudry and Meir (2012b). Specifically, due to the presence of noise, we were able to linearize the map's dynamics, and derive an explicit input–output relation. Such a linearization became possible because we made the (unusual) choice that the “input” to the CBM consists of the time-intervals between stimulation pulses, while the “output” is a binary series indicating whether or not an AP happened immediately after a stimulation pulse. The linearized input–output relation can be expressed either in biophysically interpretable “state space” (Equations 11, 12 and Figure 1C), or as a sum of the filtered input and filtered noise (Equation 20). Note that the overall I/O includes the mean output (Equation 10) which is non-linear. However, the linear part of the response, allows the derivation of the power spectral densities (Equation 13), the construction of linear optimal estimators (e.g., Figure 3C) and blind identification of the (linearized) system parameters (“Ident.” in Figures 3–5).

Our results rely on three main assumptions. The temporal sparseness of the input τ_AP_ ≪ *T*_*m*_ insures that the slow variables **s**_*m*_ effectively represent the “neuronal state” alone (as *V* and **r** always relax to a steady state before the next stimulation is given). The additional assumption *T*_*m*_ ≪ τ**_s_** allowed us to integrate the model dynamics and derive the reduced map (Equation 8) for the dynamics of **s**_*m*_, which is linear in *T*_*m*_. The last assumption is that the dynamics of **s**_*m*_ can be linearized around a stable fixed point s_*_. This fixed point is generated due to the noisiness of the rapid variables (Figure 2), and the assumption *T*_*m*_ ≪ τ**_s_** ensures that the stochastic fluctuations around s_*_ are small. We performed extensive numerical simulations (section 2.5) that indicate that our analytical results are accurate – sometimes even if our assumptions break down.

However, clearly there are cases, beyond our assumptions, in which are results cannot hold. For example, if ${\hat{T}}_{m}$ has very large fluctuations, then the response of the neuron cannot be completely linear, since $0<{\hat{Y}}_{m}<1$. Such cases may require an extension of the formalism described here. There are many possible extensions which we did not pursue here. For example, one can extend the modeling framework (e.g., multi-compartment neurons), stimulation regime (e.g., heterogeneous pulse amplitudes), or the type of neurons modeled (e.g., bursting and spontaneously firing neurons). However, it seems that an important assumption, that cannot be easily removed, is that the input is temporally sparse (τ_AP_ ≪ *T*_*m*_).

### 3.3. Practical significance

Is such a sparse temporally stimulation regime “physiologically relevant” for the soma of a neuron? Currently, such question cannot be answered directly, since it is impossible to accurately measure all the current arriving to the soma from the dendrites under completely physiological conditions. However, there is some indirect evidence. Recent studies have shown that the distribution of synaptic efficacies in the cortex is log-normal (Song et al., 2005) – so a few synapses are very strong, while most are very weak. This indicates that the neuronal firing patterns might in fact be dominated by a small number of very strong synapses while the sum of the weak synapses sets the voltage baseline (Ikegaya et al., 2012). Such a possibility is supported by the fact that individual APs can trigger the complex network events in humans (Molnár et al., 2008; Komlósi et al., 2012). Also, in rats, individual cortical cells can elicit whisker movements in Brecht et al. (2004) and even modify the global brain state (Li et al., 2009). Taken together, these observations suggests that the above-threshold stimulation reaching the soma may be temporally sparse in some cases.

There are other obvious cases were our results are immediately applicable. First, in an axonal compartment, the relevant input current is indeed sparse – an AP spike train arriving from a previous compartment. Second, a direct pulse-like stimulation is used in cochlear implants (Goldwyn et al., 2012, and references therein). Lastly, such stimulation is used as an experimental probe (De Col et al., 2008; Gal et al., 2010; Gal and Marom, 2013). Specifically, since we now have a precise expression for the power spectral density of the response, we are now ready to use these analytical results in Soudry and Meir (2014) to reproduce the experimentally observed 1/*f*^α^ behavior of the neuron and explore its implications on its input–output relation.

## 4. Methods

In this section we provide the details of the results presented in the paper. Sections 4.1–4.5 here respectively correspond to Sections 2.1–2.5. The first four (theoretical) sections can be read independently of each other (except when we discuss the repeating “HHS model” example). The last section give the details of the numerical simulations.

### 4.1. Full model (biophysical neuron models)

As we explained in section **2.1**, a general model for a biophysical isopotential neuron is given by the following equations

(24) $$\dot{V}=f\left(V,r,s,I\left(t\right)\right),$$

(25) $$\dot{r}=A_{r}\left(V\right)r+B_{r}\left(V,r\right)\xi_{r},$$

(26) $$\dot{s}=A_{s}\left(V\right)s+B_{s}\left(V,s\right)\xi_{s},$$

with **voltage** *V*, stimulation current *I*(*t*), **rapid** variables **r** (e.g., *m, n, h* in the Hodgkin–Huxley (HH) model Hodgkin and Huxley, 1952), **slow** variables **s** (e.g., slow sodium inactivation Chandler and Meves, 1970), rate matrices **A**_*r/s*_, white noise processes ξ_*r/s*_ (with zero mean and unit variance), and matrices **B**_*r/s*_ which can be written explicitly using the rates and ion channel numbers (Orio and Soudry, 2012) (**D** = **BB**^⊤^ is the diffusion matrix Gardiner, 2004; Orio and Soudry, 2012). In this section we give the specific forms of **A**_*r/s*_ and **B**_*r/s*_, and their origin based on neuronal biophysics.

#### Microscopic origins

Such a model is commonly called a stochastic Conductance Based Model (CBM). In a non-stochastic CBM, the dynamics of the membrane voltage *V* (Equation 36) are deterministically determined by some general function of *V*, the stimulation current *I*(*t*), and some internal state variables **r** and **s**. In contrast, the dynamical equations for **r** and **s** here adhere to a specific Stochastic Differential Equation (SDE) form, since these variables describe the “population state” of all the ion channels in the neuron. We now explain the biophysical interpretation of those equations.

At the microscopic level, each ion channel has several states, and it switches between those states with voltage dependent rates (Hille, 2001). This is usually modeled using a Markov model framework (Colquhoun and Hawkes, 1981). Formally, suppose we index by *c* the different types of channels, *c* = 1, …, *C*. For each channel type *c* there exist *N*^(*c*)^ channels, where each channel of type *c* possesses *K*^(*c*)^ internal states. In the Markov framework, for each ion channel that resides in state *i*, the probability that the channel will be in state *j* after an infinitesimal time *dt* is given by

(27) $$\{\begin{array}{ll} A_{ij}^{\left(c\right)}\left(V\right)dt, & \text{if }j\neq i\\ 1-\sum_{j\neq i}A_{ji}^{\left(c\right)}\left(V\right)dt, & \text{if }j=i \end{array},$$

where **A**^(*c*)^ (*V*) is called the “rate matrix” for that channel type.

To facilitate mathematical analysis and efficient numerical simulation, we preferred to model stochastic CBMs using a compressed, SDE form. This method was initially suggested by Fox and Lu (1994), but their method suffered from several problems (Goldwyn et al., 2011). In a recent paper (Orio and Soudry, 2012) a more general method was derived, which had none of the previous problems, and was shown numerically to produce a very accurate approximation of the original Markov process description.

#### Derivation

According to Orio and Soudry (2012), if we define *x*^(*c*)^_*k*_ to be the fraction of *c*-type channels in state *k*, and **x**^(*c*)^ to be a column vector composed of *x*^(*c*)^_*k*_, then

(28) $${\dot{x}}^{\left(c\right)}=A^{\left(c\right)}\left(V\right)x^{\left(c\right)}+B^{\left(c\right)}\left(V,x^{\left(c\right)}\right)\xi^{\left(c\right)},$$

where ξ^(*c*)^ is a white noise vector process – meaning it has zero mean and auto-covariance

$$\langle \xi^{\left(c\right)}\left(t\right){\left(\xi^{\left(c\right)}\left(t^{\prime }\right)\right)}^{⊤}\rangle =I\delta_{c,c^{\prime }}\delta \left(t-t^{\prime }\right)$$

where **I** is the identity matrix, δ(*t*) is the Dirac delta function, and δ_*c, c′*_ = 1 if *c* = *c′* and 0 otherwise. Furthermore, **B**^(*c*)^ is defined so that in Equation (28) each component of ξ^(*c*)^, which is associated with a transition pair *i* ⇋ *j*, is multiplied by $\sqrt{\left(A_{ij}^{\left(c\right)}x_{j}^{\left(c\right)}+A_{ji}^{\left(c\right)}x_{i}^{\left(c\right)}\right)/N^{\left(c\right)}}$, and appears in the equation for ${\dot{x}}_{i}^{\left(c\right)}$ and ${\dot{x}}_{j}^{\left(c\right)}$ with opposite signs. Note that **B**^(*c*)^ is not necessarily square since it has *K*^(*c*)^ rows but the number of columns is equal to the number of transition pairs.

We now need to combine Equation (28) for all *c* to obtain Equations (1–3). For simplicity, assume now that all ion channels types can be classified as either “rapid” or “slow” (this assumption can be relaxed). In this case we can concatenate all vectors related to rapid channels $r≜{\left(x_{\left(1\right)}^{⊤},\ldots ,x_{\left(R\right)}^{⊤}\right)}^{⊤}$ and to slow channels $s≜{\left(x_{\left(R\;+\;1\right)}^{⊤},\ldots ,x_{\left(R+S\right)}^{⊤}\right)}^{⊤}$, where *R* + *S* = *C*. We similarly define ξ_*r*_ and ξ_*s*_ together with the block matrices

$$A_{r}≜\left(\begin{array}{cccc} \begin{array}{c} A^{\left(1\right)} \end{array} & 0 & \ldots & 0\\ 0 & A^{\left(2\right)} & \ldots & 0\\ \vdots & \vdots & \ddots & \vdots \\ 0 & 0 & \cdots & A^{\left(R\right)} \end{array}\right),A_{s}≜\left(\begin{array}{cccc} A^{\left(R+1\right)} & 0 & \ldots & 0\\ 0 & A^{\left(R+2\right)} & \ldots & 0\\ \vdots & \vdots & \ddots & \vdots \\ 0 & 0 & \cdots & A^{\left(R+S\right)} \end{array}\right)$$

and similarly for **B**_*r*_ and **B**_*s*_. Note that **A**_*r*_ is square with size $\tilde{M}=\sum_{c\;=\;1}^{R}K^{\left(c\right)}$ rows while **A**_*s*_ is square with size $\tilde{M}=\sum_{c=R\;+\;1}^{R\;+\;S}K^{\left(c\right)}$ rows.

#### 4.1.1. Compressed formulation

In some cases, it is more convenient to re-write Equations (1–3) in a compressed form (this is always possible)

(29) $$\dot{V}=f\left(V,r,s,I\left(t\right)\right),$$

(30) $$\dot{r}={\tilde{A}}_{r}\left(V\right)r-b_{r}\left(V\right)+{\tilde{B}}_{r}\left(V,r\right)\xi_{r},$$

(31) $$\dot{s}={\tilde{A}}_{s}\left(V\right)s-b_{s}\left(V\right)+{\tilde{B}}_{s}\left(V,s\right)\xi_{s},$$

where **r, s**, and ξ_*r/s*_ have been redefined (their dimension has decreased), as we will show next. First, we comment that the main disadvantage is of these equations is that they are less compact and the notation is somewhat more cumbersome. However, there are also several advantages to this approach: (1) The vectors and matrices are smaller, (2) The rate and diffusion matrices do not have “troublesome” zero eigenvalues and can be diagonal (which is analytically convenient), (3) Most CBMs are written using this form (e.g., the HH model), so it is easier to apply our results using this formalism.

#### Derivation

To derive these compressed equations, we use the fact *x*^(*c*)^_*k*_ denote fractions, so ∑_*k*_*x*^(*c*)^_*k*_ = 1, for all *c*. We can use this constraint, together with the irreducibility of the underlying ion channel process, to reduce by one the dimensionality of Equation (28) (see Soudry and Meir, 2012a for further details). Defining **I** to be the identity function, **J** to be the **I** with it last row removed, **e** ≜ (0, 0, …, 1)^⊤^, **u** ≜ (1, 1, …, 1)^⊤^, **G** ≜ (**I** − **eu**^⊤^) **J**^⊤^, ${\tilde{A}}^{\left(c\right)}≜JA^{\left(c\right)}G$, ${\tilde{B}}^{\left(c\right)}≜JB^{\left(c\right)}$ (with *x*^(*c*)^_*K*^(*c*)^_ replaced by 1 − *x*_1_ − *x*_2_ … −*x*_*K*^(*c*)^−1_) and **b**^(*c*)^ ≜ −**JA**^(*c*)^**e** (${\tilde{A}}^{\left(c\right)}$ is invertible), we obtain the following equation for the reduced state vector **y**^(*c*)^ = **Jx**^(*c*)^ (which has only *K*^(*c*)^ − 1 states)

$${\dot{y}}^{\left(c\right)}={\tilde{A}}^{\left(c\right)}y^{\left(c\right)}-b+{\tilde{B}}^{\left(c\right)}\xi^{\left(c\right)}.$$

Again assuming that all channels can be classified as either “rapid” or “slow,” we concatenate all vectors related to rapid channels **r** ≜ (**y**^⊤^_(1)_, …, **y**^⊤^_(*R*)_)^⊤^ and to slow channels **s** ≜ (**y**^⊤^_(*R*+1)_, …, **y**^⊤^_(*R*+*S*)_)^⊤^, where *R* + *S* = *C*. We obtain Equations (30, 31) by similarly defining **b**_*r*_, **b**_*s*_, ξ_*r*_ and ξ_*s*_ together with the block matrices

$${\tilde{A}}_{r}≜\left(\begin{array}{c} \begin{array}{cccc} {\tilde{A}}^{\left(1\right)} & 0 & \ldots & 0\\ 0 & {\tilde{A}}^{\left(2\right)} & \ldots & 0\\ \vdots & \vdots & \ddots & \vdots \\ 0 & 0 & \cdots & {\tilde{A}}^{\left(R\right)} \end{array} \end{array}\right),{\tilde{A}}_{s}≜\left(\begin{array}{cccc} {\tilde{A}}^{\left(R+1\right)} & 0 & \ldots & 0\\ 0 & {\tilde{A}}^{\left(R+2\right)} & \ldots & 0\\ \vdots & \vdots & \ddots & \vdots \\ 0 & 0 & \cdots & {\tilde{A}}^{\left(R+S\right)} \end{array}\right)\text{​},$$

and similarly for ${\tilde{B}}_{r}$ and ${\tilde{B}}_{s}$. Note that ${\tilde{A}}_{r}$ is square with ${\tilde{M}}_{r}=\sum_{c\;=\;1}^{R}K^{\left(c\right)}-R$ rows while ${\tilde{A}}_{s}$ is square with ${\tilde{M}}_{s}=\sum_{c\;=\;R\;+\;1}^{R+S}K^{\left(c\right)}-S$ rows. Furthermore, it can be shown (Soudry and Meir, 2012a) that ${\tilde{A}}^{\left(c\right)}$ is a strictly stable matrix (all its eigenvalues are also eigenvalues of **A**^(*c*)^ except its zero eigenvalue, and so have a strictly negative real part), and ${\tilde{D}}^{\left(c\right)}≜{\tilde{B}}^{\left(c\right)}{\tilde{B}}^{\left(c\right)⊤}$ is positive definite (so all its eigenvalues are real and strictly positive). Therefore, also ${\tilde{A}}_{r}$ and ${\tilde{A}}_{s}$ are both strictly stable and ${\tilde{D}}_{r}$ and ${\tilde{D}}_{s}$ are positive definite. Therefore, if *V* is held constant, 〈**s**〉 and 〈**r**〉 tend to $s_{\infty }={\tilde{A}}_{s}^{-1}b_{s}$ and $r_{\infty }={\tilde{A}}_{r}^{-1}b_{r}$, respectively.

#### Example – the HHS model

The HHS model can be easily written using the compressed formulation. For example, comparing Equation (23) with Equation (31) we find that

(32) $$A_{s}\left(V\right)=-\gamma \left(V\right)-\delta \left(V\right)$$

(33) $$b_{s}\left(V\right)=-\delta \left(V\right)$$

(34) $$B_{s}\left(V,s\right)=\sqrt{\left(\delta \left(V\right)\left(1-s\right)+\gamma \left(V\right)s\right)N_{s}^{-1}\phi }$$

(35) $$D_{s}\left(V,s\right)=\left(\delta \left(V\right)\left(1-s\right)+\gamma \left(V\right)s\right)N_{s}^{-1}\phi .$$

Note that all the parameters are scalar in the HHS model, and so are not boldfaced, as in the general case.

### 4.2. Model reduction

In this section we give additional technical details on section **2.2**. Specifically, we show how, given sparse spike stimulation and a few assumptions, it is possible to derive a simple reduced dynamical system (Equation 8) from the full equations of a general biophysical model for an isopotential neuron (Equations 1–3),

(36) $$\dot{V}=f\left(V,r,s,I\left(t\right)\right),$$

(37) $$\dot{r}=A_{r}\left(V\right)r+B_{r}\left(V,r\right)\xi_{r},$$

(38) $$\dot{s}=A_{s}\left(V\right)s+B_{s}\left(V,s\right)\xi_{s}.$$

For more details on how its parameters and variables map to microscopic biophysical quantities, see section 4.1.

#### 4.2.1. The excitability constraint

As explained in section 2.1, we focus on models for **excitable** neurons describable by equations of the general form of Equations (36–38), rather than on arbitrary dynamical systems. This imposes some constraints on the parameters (Soudry and Meir, 2012b). Formally, recall that τ_AP_ and τ**_s_** are the respective kinetic timescales of {*V*, **r**} and **s**, and that τ_AP_ < τ**_s_**. Suppose we “freeze” the dynamics of **s** (so that effectively τ_**s**_ = ∞) and allow only *V* and **r** to evolve in time. We say the original model describes an **excitable** neuron, if the following conditions hold in this “semi-frozen” model:

1. If *I*(*t*) = 0, then for all initial conditions, *V* and **r** rapidly (within timescale τ_AP_) relax to a constant and unique steady state (“rest”).
2. Assume that *V* and **r** are near rest, and a short stimulation pulse is given with duration *t*_stim_ ≤ τ_AP_ and amplitude *I*_0_. For certain initial conditions and values of *I*_0_, we get either a stereotypical “strong” response (“AP response”) or a stereotypical “weak” response in *V* (“no AP response”). Only for a very small set of initial conditions and values of *I*_0_, do we get an “intermediate” response (“weak AP response”). By “stereotypical” we mean that the shape of response does not change much between trials or for different initial conditions in {*V*, **r**} (however, it can change with **s**).

Note that due to condition 1, such an excitable neuron is *not* oscillatory and does not spontaneously fire APs.

#### 4.2.2. Problem formulation

Formally, suppose an excitable neuron receives a train of *identical* stimuli, so

$$I\left(t\right)=\sum_{m=-\infty }^{\infty }⊓\left(t-t_{m}\right),$$

where ⊓(*x*) is a pulse, of width *t*_*w*_ (so ⊓(*x*) = 0 for *x* outside [0, *t*_*w*_]). We denote by {*Y*_*m*_}^∞^_*m*=−∞_ the occurrence events of AP responses at times {*t*_*m*_}^∞^_*m*=−∞_, i.e., immediately after each stimulation time *t*_*m*_ (Figure 1A),

(39) $$Y_{m}≜\{\begin{array}{l} \begin{array}{lll} 1 & , & \text{if an AP occurs}\\ 0 & , & \text{otherwise} \end{array} \end{array}.$$

Defining *T*_*m*_ ≜ *t*_*m* + 1_ − *t*_*m*_, the inter-stimulus interval, and τ_AP_ as the upper timescale of an AP event (Figure 1B) we make the following assumption.

**Assumption 1**. *(a) The stimulation pulse width is small, *t*_*w*_ < τ_AP_. (b) The spike times* {*t*_*m*_}^∞^_*m* = 0_ *are temporally sparse, i.e*., τ_AP_ ≪ *T*_*m*_ *for every m (“no collisions”)*.

Our main objective here is to mathematically characterize the relation between {*Y*_*m*_} and {*T*_*m*_} under the most general conditions.

#### 4.2.3. Derivations

We define the sampled quantities *V*_*m*_ ≜ *V*(*t*_*m*_), **r**_*m*_ ≜ **r**(*t*_*m*_), **s**_*m*_ ≜ **s**(*t*_*m*_), **x**_*m*_ ≜ (*V*_*m*_, **r**^⊤^_*m*_, **s**^⊤^_*m*_)^⊤^ and the history set 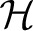
_*m*_ ≜ $\left\{{\left\{x_{k}\right\}}_{k=-\infty }^{m},{\left\{T_{k}\right\}}_{k=-\infty }^{m},{\left\{Y_{k}\right\}}_{k=-\infty }^{m}\right\}$ (note that 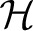
_*m*_ ⊂ 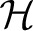
_*m* + 1_). The Stochastic Differential Equation (SDE) description in Equations (36–38) implies that **x**_*m*_ is a “state vector” with the *Markov property*, namely it is a sufficient statistic on the history to determine the probability of generating an AP at each stimulation,

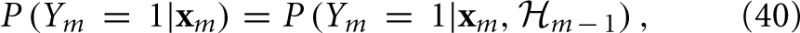



and, together with *Y*_*m*_ and *T*_*m*_, its own dynamics

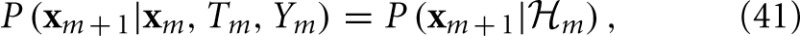



which implies the following causality relations

(42) $$\begin{array}{ccccccc} x_{0} & \overset{T_{0}}{\to } & x_{1} & \overset{T_{1}}{\to } & x_{2} & \cdots & x_{m}\\ ↓ & ↗ & ↓ & ↗ & ↓ & & ↓\\ Y_{0} & & Y_{1} & & Y_{2} & \cdots & Y_{m} \end{array}.$$

This causality structure is reminiscent of the well known Hidden Markov Model (Rabiner, 1989), except that in the present case the output *Y*_*m*_, affects the transition probability, and we have input *T*_*m*_. Theoretically, if we knew the distributions in Equations (40) and (41), as well as the initial condition *P* (**x**_0_), we could integrate and find an exact probabilistic I/O relation *P*({*Y*_*k*_}^*m*^_*k*=*0*_|{*T*_*k*_}^*m*^_*k*=0_. However, since it may be hard to find an expression for *P*(**x**_*m* + 1_|**x**_*m*_, *T*_*m*_, *Y*_*m*_) in general, we make a simplifying assumption.

**Assumption 2**. *T*_*m*_ ≪ *τ_s_ for every m*.

This assumption, together with Assumption 1 and the excitable nature of the CBM, renders the dynamics between stimulations relatively easy to understand. Specifically, between two consecutive stimulations, the fast variables (*V*(*t*), **r**(*t*)) follow stereotypically either the “AP response” (*Y*_*m*_ = 1) or the “no-AP response” (*Y*_*m*_ = 0), then equilibrate rapidly (within time τ_AP_) to some quasi-stationary distribution *q*(*V*, **r**|**s**_*m*_). Meanwhile, the slow variable **s** (*t*), starting from its initial condition at the time of the previous stimulation, changes slowly according to Equation (38), affected by the voltage trace of *V*(*t*) (through **A**_*s*_ (*V*)).

Summarizing this mathematically, we obtain the following approximations

(43) $$P\left(Y_{m}|s_{m}\right)\approx \int P\left(Y_{m}|V,r,s_{m}\right)q\left(V,r|s_{m}\right)dVdr,$$

(44) $$\begin{array}{l} P\left(V_{m+1},r_{m+1},s_{m+1}|s_{m},T_{m},Y_{m}\right)\approx q\left(V_{m+1},r_{m+1}|s_{m+1}\right)\\ P\left(s_{m+1}|s_{m},T_{m},Y_{m}\right). \end{array}$$

Using these equations together with Equations (40) and (41), we obtain

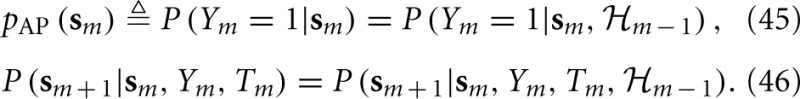



Therefore, the “excitability” vector **s**_*m*_ can now replace the full state vector **x**_*m*_ = (*V*_*m*_, **r**^⊤^_*m*_, **s**^⊤^_*m*_)^⊤^ as the sufficient statistic that retains all relevant the information about the history of previous stimuli. Given the input {*T*_*m*_}^∞^_*m*=−∞_, Equations (45) and (46) together completely specify a Markov process with the causality structure

(47) $$\begin{array}{ccccccc} \begin{array}{c} s_{0} \end{array} & \overset{T_{0}}{\to } & s_{1} & \overset{T_{1}}{\to } & s_{2} & \cdots & s_{m}\\ ↓ & ↗ & ↓ & ↗ & ↓ & & ↓\\ Y_{0} & & Y_{1} & & Y_{2} & \cdots & Y_{m} \end{array}.$$

Since the function *p*_AP_ (**s**) is not affected by the kinetics of **s**, it can be found by numerical simulation of a single AP using only Equations (36, 37), when **s** is held constant (see section 4.2.4). Now, instead of finding *P*(**s**_*m* + 1_|**s**_*m*_, *Y*_*m*_, *T*_*m*_) directly, we calculate the increments Δ**s**_*m*_ ≜ **s**_*m* + 1_ − **s**_*m*_ by integration of the SDE in Equation (38) between *t*_*m*_ and *t*_*m* + 1_. First, we integrate the “predictable” part of the increment

(48) $$\langle \Delta s_{m}|s_{m},T_{m},Y_{m}\rangle =\int_{t_{m}}^{t_{m+1}}A_{s}\left(V\left(t\right)\right)s\left(t\right)dt,$$

(49) $$\;\approx \left(\int_{t_{m}}^{t_{m+1}}A_{s}\left(V\left(t\right)\right)dt\right)s_{m},$$

to first order, where 〈*X*|*Y*〉 denotes the conditional expectation of *X* given *Y*. Note that **A**_*s*_ ~ *O*(τ**_s_**^−1^), so second order corrections are of order *O*((*T*_*m*_τ**_s_**^−1^)^2^). Due to assumption 2, we have *T*_*m*_τ**_s_**^−1^ ≪ 1, so these corrections are negligible. Now,

$$\begin{array}{l} \int_{t_{m}}^{t_{m+1}}A_{s}\left(V\left(t\right)\right)dt=\tau_{\text{AP}}\left(\frac{1}{\tau_{\text{AP}}}\int_{t_{m}}^{t_{m}+\tau_{\text{AP}}}\text{​}A_{s}\left(V\left(t\right)\right)dt\right)\\ \;+\text{​}\left(T_{m}-\tau_{\text{AP}}\right)\text{​}\left(\frac{1}{T_{m}-\tau_{\text{AP}}}\text{​}\int_{t_{m}+\tau_{\text{AP}}}^{t_{m+1}}A_{s}\left(V\left(t\right)\right)dt\right)\\ \;=\tau_{\text{AP}}\left(A_{+}\left(s_{m}\right)Y_{m}+A_{-}\left(s_{m}\right)\left(1-Y_{m}\right)\right)\\ \;+\left(T_{m}-\tau_{\text{AP}}\right)A_{0}\left(s_{m}\right) \end{array}$$

where we defined

(50) $$A_{0}\left(s_{m}\right)=\frac{1}{T_{m}-\tau_{\text{AP}}}\int_{t_{m}+\tau_{\text{AP}}}^{t_{m+1}}A_{s}\left(V\left(t\right)\right)dt,$$

(51) $$A_{-}\left(s_{m}\right)=\frac{1}{\tau_{\text{AP}}}\int_{t_{m}}^{t_{m}+\tau_{\text{AP}}}A_{s}\left(V\left(t\right)\right)dt,\text{ if }Y_{m}=0,$$

(52) $$A_{+}\left(s_{m}\right)=\frac{1}{\tau_{\text{AP}}}\int_{t_{m}}^{t_{m}+\tau_{\text{AP}}}A_{s}\left(V\left(t\right)\right)dt,\text{ if }Y_{m}=1,$$

which are the average rates during rest, during an AP response and during a no-AP response, receptively. Note a similar notation was also used in Soudry and Meir (2012b) (e.g., Equations 2.15, 2.16 there), where the +/ − /0 were replaced with *H*/*M*/*L*.

Next, we calculate the remaining part of the increment, which is the “innovation,”

$$n_{m}≜\Delta s_{m}-\langle \Delta s_{m}|s_{m},T_{m},Y_{m}\rangle .$$

Obviously, 〈**n**_*m*_|**s**_*m*_, *T*_*m*_, *Y*_*m*_〉 = 0, and also

$$\begin{array}{l} \;\langle n_{m}n_{m}^{⊤}|s_{m},T_{m},Y_{m}\rangle =\langle \left(\int_{t_{m}}^{t_{m+1}}B_{s}\left(V\left(t\right),s\left(t\right)\right)\xi_{s}\left(t\right)dt\right)\\ \;(\int_{t_{m}}^{t_{m+1}}B_{s}\left(V\left(t\right),s\left(t^{\prime }\right)\right){\xi_{s}\left(t^{\prime }\right)dt^{\prime })}^{⊤}|s_{m},T_{m},Y_{m}\rangle \\ =\int_{t_{m}}^{t_{m+1}}dt\text{​}\int_{t_{m}}^{t_{m+1}}dt^{\prime }\delta \text{​}\left(t-t^{\prime }\right)\text{​}B_{s}\text{​}\left(V\left(t\right),s\text{​}\left(t\right)\right)B_{s}^{⊤}\left(V\left(t^{\prime }\right),s\left(t^{\prime }\right)\right)\\ =\int_{t_{m}}^{t_{m+1}}B_{s}\left(V\left(t\right),s\left(t\right)\right)B_{s}^{⊤}\left(V\left(t^{\prime }\right),s\left(t\right)\right)dt\\ =\int_{t_{m}}^{t_{m+1}}D_{s}\left(V\left(t\right),s\left(t\right)\right)dt\\ \approx \int_{t_{m}}^{t_{m+1}}D_{s}\left(V\left(t\right),s_{m}\right)dt \end{array}$$

to first order. Note that **D**_*s*_ ~ *O*(τ**_s_**^−1^/*N*), where *N* = min_*c*_*N*^(*c*)^(*N*^(*c*)^ is the channel number of the *c*-type channel, as we defined in section 4.1), while Equation (53) has corrections of size *O*((*T*_*m*_τ**_s_**^−1^/*N*)^2^). Since *N* ≥ 1 (usually *N* ≫ 1), and due to assumption 2, we have *T*_*m*_τ**_s_**^−1^/*N* ≪ 1, so these corrections are also negligible. Now,

(53) $$\begin{array}{l} \;\int_{t_{m}}^{t_{m+1}}D_{s}\left(V\left(t\right),s_{m}\right)dt\\ =\tau_{\text{AP}}\left(Y_{m}D_{+}\left(s_{m}\right)+\left(1-Y_{m}\right)D_{-}\left(s_{m}\right)\right)+\left(T_{m}-\tau_{\text{AP}}\right)D_{0}\left(s_{m}\right) \end{array}$$

where we defined

(54) $$D_{0}\left(s_{m}\right)=\frac{1}{T_{m}-\tau_{\text{AP}}}\int_{t_{m}+\tau_{\text{AP}}}^{t_{m+1}}D_{s}\left(V\left(t\right),s_{m}\right)dt$$

(55) $$D_{-}\left(s_{m}\right)=\frac{1}{\tau_{\text{AP}}}\int_{t_{m}}^{t_{m}+\tau_{\text{AP}}}D_{s}\left(V\left(t\right),s_{m}\right)dt,\text{ if}Y_{m}=0$$

(56) $$D_{+}\left(s_{m}\right)=\frac{1}{\tau_{\text{AP}}}\int_{t_{m}}^{t_{m}+\tau_{\text{AP}}}D_{s}\left(V\left(t\right),s_{m}\right)dt,\text{ if }Y_{m}=1.$$

Additionally, we note that **A**_±/0_ (**s**_*m*_) generally tend to be rather insensitive to changes in **s**_*m*_. This is because the kinetic transition rates (which are used to construct **A**_*s*_ (*V*), as explained in section 4.1) tend to demonstrate this insensitivity when similarly averaged (see Figures 4B, 5 in Soudry and Meir, 2012b). The usual reasons behind this are (see appendix section B1 of Soudry and Meir, 2012b): (1) The common sigmoidal shape of the voltage dependency of the kinetic rates reduces their sensitivity to changes in the amplitude of the AP or the resting potential (2) The shape of the AP is relatively insensitive to **s** (3) The resting voltage is relatively insensitive to **s**. Therefore, in most cases we can approximate **A**_±/0_ (**s**_*m*_) to be constant for simplicity (though this not critical to our subsequent results), as we shall henceforth do.

Additionally, we note that, strictly speaking, the voltage trace during an AP and at rest are stochastic, and therefore, **A**_+_, **A**_−_, **A**_0_, **D**_+_, **D**_−_ and **D**_0_ are stochastic. However, there are two factors that render this stochasticity negligible. First, the sigmoidal shape of the kinetic rates implies that **A**(*V*) is rather insensitive to fluctuations in the voltage (Figure 4 in our Soudry and Meir, 2012b). Second, noise mainly plays a role in the timing of AP initiation, but does not much affect the AP shape above threshold (see AP voltage traces in Schneidman et al., 1998, p. 1687). Therefore, we shall approximate **A**_+_, **A**_−_, **A**_0_, **D**_+_, **D**_−_ and **D**_0_ to be deterministic.

In summary, defining

(57) $$\begin{array}{l} A\left(Y_{m},T_{m}\right)=\tau_{\text{AP}}T_{m}^{-1}\left(Y_{m}A_{+}+\left(1-Y_{m}\right)A_{-}\right)+\\ \;\left(1-\tau_{\text{AP}}T_{m}^{-1}\right)A_{0}, \end{array}$$

and

(58) $$\begin{array}{l} D\left(Y_{m},T_{m},s_{m}\right)=\tau_{\text{AP}}T_{m}^{-1}\left(Y_{m}D_{+}\left(s_{m}\right)+\left(1-Y_{m}\right)D_{-}\left(s_{m}\right)\right)\\ \;+\left(1-\tau_{\text{AP}}T_{m}^{-1}\right)D_{0}\left(s_{m}\right) \end{array}$$

we can write

(59) $$\Delta s_{m}=T_{m}A\left(Y_{m},T_{m}\right)s_{m}+n_{m},$$

with〈**n**_*m*_|**s**_*m*_, *T*_*m*_, *Y*_*m*_〉 = 0 and

(60) $$\langle n_{m}n_{m}^{⊤}|s_{m},T_{m},Y_{m}\rangle =T_{m}D\left(Y_{m},T_{m},s_{m}\right).$$

These equations correspond to the result presented in Equation (8).

Finally, we note that the distribution of **n**_*m*_ given **s**_*m*_, *T*_*m*_, *Y*_*m*_ can be generally computed using the approach described in Orio and Soudry (2012). For example, it can be well approximated to have a normal distribution if channel numbers are sufficiently high and channel kinetics are not too slow (Orio and Soudry, 2012). In that case only knowledge of the variance (Equation 60) is sufficient to generate **n**_*m*_. And so, using Equations (45), (59) and the full distribution of **n**_*m*_, we can now simulate the neuronal response using a reduced model, more efficiently and concisely (with fewer parameters) than the full model (Equations 36, 38), since every time step is a stimulation event. The simulation time should shorten approximately by a factor of 〈*T*_*m*_〉/*dt*, where *dt* is the full model simulation step. Note that the reduced model parameters, having been deduced from the full model itself, still retain a biophysical interpretation.

#### 4.2.4. Calculation of *p*_AP_ (**s**)

We numerically calculated *p*_AP_ (**s**) by disabling all the slow kinetics in the model – i.e., we only use Equations (1, 2) in main text, while **ṡ** = 0. Then, for every value of **s** we simulated this “semi-frozen” model numerically by first allowing **r** to relax to a steady state and then giving a stimulation pulse with amplitude *I*_0_. We repeat this procedures 200 times for each **s**, and calculate *p*_AP_ (**s**) as the fraction of simulations that produced an AP. A few comments are in order: (1) In some cases (e.g., the HHMS model) we can use a shortcut and calculate *p*_AP_ (**s**) based on previous results. For example, suppose we know the probability function $\tilde{p}$_AP_ (*s*) for some model with a scalar ***s*** and we make the substitution *s* = *h* (**s**) where the components of **s** represent independent and uncoupled channel types (Orio and Soudry, 2012) – then *p*_AP_ (**s**) = $\tilde{p}$_AP_ (*h* (**s**)) in the new model. (2) The timescale separation assumption τ_AP_ ≪ *T*_*m*_ ≪ τ**_s_** implies that all the properties of the generated AP (amplitude, latency etc.) maintain similar causality relations with **s**_*m*_ as does *Y*_*m*_, so we can find their distribution using the same simulation we used to find *p*_AP_ (**s**), similarly to the approach taken to compute *L* (**s**) in the deterministic setting (Soudry and Meir, 2012b). (3) Numerical results (Figure 6) suggest that we can generally write

(61) $$p_{\text{AP}}\left(s\right)=\Phi \left(E\left(s\right)/\sqrt{N_{r}}\right),$$

where Φ is the cumulative distribution function of the normal distribution, *E* (**s**) is some “excitability function” (as defined in Soudry and Meir (2012b), so *p*_AP_ (**s**) = 0.5 on the threshold Θ = {**s**|*E* (**s**) = 0}), and *N*^−1/2^_*r*_, the “noisiness” of the rapid sub-system, directly affects the slope of *p*_AP_ (**s**) (Figure 6D, *bottom*). Also, as explained in Soudry and Meir (2012b), *E* (**s**) is usually monotonic in each component separately and increasing in *I*_0_ (Figure 6C, *top*) – which could be considered as just another component of ****s**** which has zero rates.

#### 4.2.5. Compressed formulation – reduction

We can perform a very similar model reduction and linearization using the compressed formalism presented in section 4.1.1. We just need to define (or re-define) **A**_±,0_, **b**_±,0_, **D**_±,0_ (**s**_*m*_), **A** (*Y*_*m*_, *T*_*m*_), **b** (*Y*_*m*_, *T*_*m*_) and **D** (*Y*_*m*_, *T*_*m*_, **s**_*m*_) in the obvious way and repeat very similar derivations, arriving to

$$\Delta s_{m}=T_{m}\left[A\left(Y_{m},T_{m}\right)s_{m}-b\left(Y_{m},T_{m}\right)\right]+n_{m},$$

instead of Equation (59) (or Equation 8). Next, we demonstrate this for the HHS model.

#### 4.2.6. Example – HHS model reduction

We derive the parameters of the HHS reduced map. Recall that the HHS model is based on the compressed formulation. Following the reduction technique described in the previous sections, we numerically find the average rates γ_±,0_ and δ_±,0_ (as in Equations (2.15, 2.16) of Soudry and Meir (2012b), where there we denoted *H*/*M*/*L* instead of +/ − /0 here), τ_AP_ and *p*_AP_ (*s*) (section 4.2.4).

From Equations (32, 35), we find,

(62) $$A_{\pm ,0}=-\gamma_{\pm ,0}-\delta_{\pm ,0}$$

(63) $$b_{\pm ,0}=-\delta_{\pm ,0}$$

(64) $$D_{\pm ,0}\left(s\right)=N_{s}^{-1}\left(\delta_{\pm ,0}\left(1-s\right)+\gamma_{\pm ,0}s\right).$$

and so *A* (*Y*_*m*_, *T*_*m*_) and *D* (*Y*_*m*_, *T*_*m*_, *s*_*m*_) are defined as in Equations (57) and (58), and similarly

$$b\text{​}\left(Y_{m},T_{m}\right)\text{​}=\text{​}\tau_{\text{AP}}T_{m}^{-1}\text{​}\left(Y_{m}b_{+}+\left(1-Y_{m}\right)b_{-}\right)+\text{​}\left(1-\tau_{\text{AP}}T_{m}^{-1}\right)b_{0}.$$

We give for example some specific values: if τ_AP_ = 15 ms, then in the range *I*_0_ = 7.5−8.3 μ*A*, we have δ_±,0_ = 25.5−25.6 mHz, γ_+_ = 22.9−22.1 mHz, γ_−_ = 0.9−1.3μ Hz and γ_0_ = 0.29−0.28μ Hz.

Recall that these averaged kinetic rates are determined by the shape of the voltage dependent rates (γ (*V*) and δ (*V*), see Equation 125) (Soudry and Meir, 2012b). The relative values of the averaged kinetic rates determine what kind of information can be stored in *s* (which retains the “memory” of the neuron between stimulation). We qualitatively demonstrate this in Figure 7 depicting the values of γ_±,0_ for three different shapes of γ (*V*): when γ (*V*) is sigmoidal with high threshold, when it is sigmoidal with low threshold and when it is constant. These determine whether γ (*V*) is affected by the output (APs), the input (stimulation pulses) or neither. Therefore: (1) if γ (*V*) and δ (*V*) are independent of the voltage, then *s* cannot store any information on input or output. (2) if γ (*V*) or δ (*V*) have low voltage threshold, then *s* can directly store information on the input. (3) if γ (*V*) or δ (*V*) have high voltage threshold, then *s* can directly store information about the output. In the HHS model the inactivation rate γ has high threshold, while δ is voltage independent – therefore, *s* directly stores information on the output.

### 4.3. Linearization

In this section we present a more detailed account on how to arrive from the reduced model (mainly, Equation 8) to its linearized version (the results in Equations 11, 12).

First, we write the complete reduced model, using Equations (59), (60), and (45). The reduced model is a non-linear stochastic dynamic “state-space” system with *T*_*m*_, the inter-stimulus interval lengths, serving as inputs, **s**_*m*_ representing the neuronal state, and *Y*_*m*_ the output. We have

(65) $$\Delta s_{m}=T_{m}A\left(Y_{m},T_{m}\right)s_{m}+n_{m},$$

(66) $$Y_{m}=p_{\text{AP}}\left(s_{m}\right)+e_{m},$$

where 〈**n**_*m*_**n**^⊤^_*m*_|**s**_*m*_, *T*_*m*_, *Y*_*m*_〉 = *T*_*m*_**D** (*Y*_*m*_, *T*_*m*_, **s**_*m*_),

$$\begin{array}{l} A\left(Y_{m},T_{m}\right)=\tau_{\text{AP}}T_{m}^{-1}\left(Y_{m}A_{+}+\left(1-Y_{m}\right)A_{-}\right)+\\ \;\left(1-\tau_{\text{AP}}T_{m}^{-1}\right)A_{0}, \end{array}$$

and

$$\begin{array}{l} D\left(Y_{m},T_{m},s_{m}\right)=\tau_{\text{AP}}T_{m}^{-1}\left(Y_{m}D_{+}\left(s_{m}\right)+\left(1-Y_{m}\right)D_{-}\left(s_{m}\right)\right)+\\ \;\left(1-\tau_{\text{AP}}T_{m}^{-1}\right)D_{0}\left(s_{m}\right), \end{array}$$

and we defined

(67) $$e_{m}≜Y_{m}-p_{\text{AP}}\left(s_{m}\right).$$

Based on the causality structure in Equation (42), it is straightforward to prove that *e*_*m*_ and **n**_*m*_ are uncorrelated white noise processes – i.e., 〈*e*_*m*_〉 = 0, 〈**n**_*m*_〉 = 〈*e*_*n*_**n**_*m*_〉 = **0** and 〈**n**_*m*_**n**^⊤^_*n*_〉 = 〈**n**_*m*_**n**^⊤^_*m*_〉 δ_*mn*_, 〈*e*_*m*_*e*_*n*_〉 = 〈*e*^2^_*m*_〉 δ_*mn*_ where δ_*nm*_ = 1 if *n* = m and 0 otherwise.

We now examine the case where {*T*_*m*_} is a Wide Sense Stationary (WSS) process (i.e., the first and second order statistics of the process are invariant to time shifts), with mean T_*_, so that the assumptions τ_AP_ ≪ *T*_*m*_ ≪ τ**_s_** are fulfilled with high probability. In this case the processes {**s**_*m*_} and {*Y*_*m*_} are also WSS, with constant means 〈**s**_*m*_〉 = s_*_ and 〈*Y*_*m*_〉 = p_*_. Also, it is straightforward to verify that $\langle {\hat{T}}_{m}n_{n}\rangle =0$, and $\langle {\hat{T}}_{m}e_{n}\rangle =0$.

In order to linearize the system in Equations (59–66) we denote ${\hat{T}}_{m}≜T_{m}-T_{*}$, ${\hat{Y}}_{m}≜Y_{m}-p_{*}$, ${\hat{s}}_{m}≜s_{m}-s_{*}$, $w≜{\nabla p_{\text{AP}}|}_{s^{*}}$. In order for this linearization to be accurate we require that ${\hat{s}}_{m}$ is “small enough.”

**Assumption 3**. *With high probability* $\left|{\hat{s}}_{m}\right|\ll \left|s_{*}\right|$ *(component-wise) and* $\left|w^{⊤}{\hat{s}}_{m}\right|\gg \left|{\hat{s}}_{m}^{⊤}\left({\nabla \nabla p_{\text{AP}}|}_{s^{*}}\right){\hat{s}}_{m}\right|$.

This assumption essentially means that s_*_ = s_*_(p_*_, T_*_) is a stable fixed point of the system (Equations 59–66), and stochastic fluctuations around it are small, compared to the size of the region {**s**|*p*_AP_ (**s**_*m*_) ≠ 0, 1} (usually determined by the noise level of the rapid system {*V*, **r**}, see section 4.2.4). Note that the region is usually rather narrow (Figure 6) and therefore $\left|{\hat{s}}_{m}\right|\ll \left|s_{*}\right|$ is often implied by this description. Given Assumption 3, we can approximate to first order

(68) $$p_{\text{AP}}\left(s_{m}\right)\approx p_{*}+w^{⊤}{\hat{s}}_{m},$$

which allows us to linearize Equation (66). This essentially means that the components of ${\hat{s}}_{m}$ determine the neuronal response linearly, with the components of **w** serving as the effective weights (related to the relevant conductances in the original full neuron model).

Next, we wish to linearize Equation (59). Using our assumptions, we obtain to first order

(69) $$\begin{array}{l} {\hat{s}}_{m+1}\approx {\hat{s}}_{m}+A\left(p_{*},T_{*}\right)\left(s_{*}+{\hat{s}}_{m}\right)\\ \;+A_{0}\left(s_{*}+{\hat{s}}_{m}\right){\hat{T}}_{m}+\tau_{\text{AP}}\left(A_{+}-A_{-}\right)\left(s_{*}+{\hat{s}}_{m}\right){\hat{Y}}_{m}+n_{m} \end{array}$$

Taking expectations and using Equations (66) and (68), we obtain

(70) $$0=\langle s_{m+1}-s_{m}\rangle \approx T_{*}A\left(p_{*},T_{*}\right)s_{*},$$

to zeroth order. Defining the solution of this equation is s_*_(p_*_, T_*_) and we can find p_*_ implicitly from

(71) $$p_{*}=p_{\text{AP}}\left(s_{*}\left(p_{*},T_{*}\right)\right).$$

We write the explicit solution of this equation as p_*_(T_*_). Next, using $\left|{\hat{s}}_{m}\right|\ll \left|s_{*}\right|$, Equation (70) and defining

(72) $$F≜I+T_{*}A_{*}\left(p_{*},T_{*}\right)$$

(73) $$d≜A_{0}s_{*}$$

(74) $$a≜\tau_{\text{AP}}\left(A_{+}-A_{-}\right)s_{*}$$

we can approximate Equation (69) as

(75) $${\hat{s}}_{m+1}=F{\hat{s}}_{m}+d{\hat{T}}_{m}+a{\hat{Y}}_{m}+n_{m},$$

which, together with

(76) $${\hat{Y}}_{m}=w^{⊤}{\hat{s}}_{m}+e_{m},$$

yields a simple linear state space representation with ${\hat{T}}_{m}$ as the input, ${\hat{s}}_{m}$ as the state, ${\hat{Y}}_{m}$ as the output and two uncorrelated white noise sources with variances

(77) $$\Sigma_{n}≜\langle n_{m}n_{m}^{⊤}\rangle =T_{*}D_{*}\left(p_{*},T_{*},s_{*}\right),$$

(78) $$\sigma_{e}^{2}≜\langle e_{m}^{2}\rangle \approx p_{*}-p_{*}^{2},$$

to first order.

#### 4.3.1. Derivation of w

From Equation (61), we note that generally we can write

(79) $$w=\nabla p_{\text{AP}}{\left(s\right)}_{s=s_{*}}=\frac{\nabla E\left(s_{*}\right)}{\sqrt{2\pi N_{r}}}\exp\left(-\frac{E^{2}\left(s_{*}\right)}{2N_{r}}\right),$$

where in many cases the excitability function *E* (**s**) has the form *E* (**s**) = ***μ***^⊤^**s** − θ, where the components of ***μ*** are proportional to the relevant conductances (Soudry and Meir, 2012b). Therefore, if

$$p_{*}=p_{\text{AP}}\left(s_{*}\right)=\Phi \left(E\left(s\right)/\sqrt{N_{r}}\right)\to 0\text{ or }1$$

then *E* (**s**) → ±∞, so in this case (assuming *E* (s_*_) is not a particularly “pathological” function) we have

(80) w→0.

#### 4.3.2. Compressed formulation – linearization

In the compressed formulation (introduced in sections 4.1.1 and 4.2.5), we can perform similar linearization by re-defining **F** ≜ **I** + T_*_A (p_*_, T_*_), **d** ≜ **A**_0_s_*_ − **b**_0_, **a** ≜ τ_AP_ ((**A**_+_ − **A**_−_)s_*_ − (**b**_+_ − **b**_−_)), and repeat very similar derivations, where now we can write more explicitly

(81) $$s_{*}=A^{-1}\left(p_{*},T_{*}\right)b\left(p_{*},T_{*}\right),$$

instead of Equation (70).

#### 4.3.3. Example – HHS model linearization

Note again that all the parameters are scalar now, and so are not boldfaced, as in the general case. From Equations (71) and (81) we obtain s_*_ and p_*_ for a given T_*_. Once s_*_ is known, from Equation (79) *w* can be obtained^7^. Next, we denote the average inactivation rate at steady state by

$$\gamma_{*}≜\left(p_{*}\gamma_{+}+\left(1-p_{*}\right)\gamma_{-}\right)\tau_{\text{AP}}T_{*}^{-1}+\left(1-\tau_{\text{AP}}T_{*}^{-1}\right)\gamma_{0},$$

and similarly for the recovery rate δ_*_. And so, s_*_ = δ_*_/(γ_*_ + δ_*_), and

(82) $$A_{*}=A_{*}\left(p_{*},s_{*}\right)=-\gamma_{*}-\delta_{*},$$

(83) $$b_{*}=-\delta_{*},$$

(84) $$D_{*}=D_{*}\left(p_{*},T_{*},s_{*}\right)=N_{s}^{-1}\left(\delta_{*}\gamma_{*}/\left(\gamma_{*}+\delta_{*}\right)\right).$$

Denoting γ_1_ ≜ γ_+_ − γ_−_ and similarly for δ_1_, we obtain

(85) $$F=1-T_{*}\left(\gamma_{*}+\delta_{*}\right)$$

(86) $$a=\tau_{\text{AP}}\left(\gamma_{*}\delta_{1}-\gamma_{1}\delta_{*}\right)/\left(\gamma_{*}+\delta_{*}\right)$$

(87) $$d=\left(\gamma_{*}\delta_{0}-\gamma_{0}\delta_{*}\right)/\left(\gamma_{*}+\delta_{*}\right)$$

Finally, from Equations (77, 78) we find

(88) $$\Sigma_{n}=T_{*}D_{*}$$

(89) $$\sigma_{e}^{2}=p_{*}-p_{*}^{2}.$$

### 4.4. Linear systems analysis

In section 2.4 we describe the neuronal dynamics using a linear system for the fluctuations, as depicted in Figure 1. This linear description allows us to use standard engineering tools to analyze the system. In this section we provide an easy to follow description on how this was done, for those unfamiliar with these topics.

#### 4.4.1. Second order statistics and linear systems

We start with a short reminder on some known results for stochastic processes (Papoulis and Pillai, 1965; Gardiner, 2004); these results are standard but are provided for completeness. These results will be used in later sections.

Assume {**x**_*m*_} and {**y**_*m*_} are two real-valued vector stochastic processes that are jointly wide-sense stationary (i.e., a simultaneous time shift of both processes will not change their first and second order statistics). We define the cross-covariance (recall that $\hat{x}=x-\langle x\rangle$)

$$R_{xy}\left(k\right)≜\langle {\hat{x}}_{m}{\hat{y}}_{m+k}^{⊤}\rangle$$

and the Cross-Power Spectral Density (CPSD), given by its Fourier transform

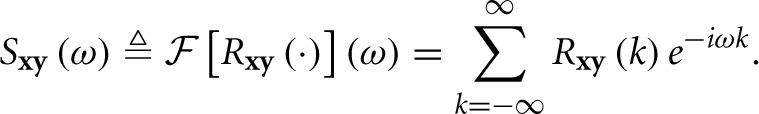



Additionally, the auto-covariance is defined as *R*_**x**_ ≜ *R*_**xx**_ and the corresponding Power Spectral Density (PSD) as *S*_**x**_ ≜ *S*_**xx**_. Also, note that *R*_**yx**_(*k*) = *R*_**xy**_^⊤^(−*k*) and so *S*_**yx**_(*k*) = *S*_**xy**_^⊤^(−ω).

Suppose now that {**y**_*m*_} is generated from a process {**x**_*m*_} using a linear system: i.e., if the Fourier transform *x*(ω) ≜ ∑^∞^_*k* = −∞_ *x*_*k*_*e*^−*i*ω*k*^ exists, then in the frequency domain

y(ω)=H(ω)x(ω),

where **H**(ω) is a matrix-valued “transfer” function. Therefore, under some regularity conditions (allowing us to switch the order of integration end expectation),

(90) $$\begin{array}{l} S_{xy}\left(\omega \right)=\sum_{k=-\infty }^{\infty }\langle {\hat{x}}_{m}{\hat{y}}_{m+k}^{⊤}\rangle e^{-i\omega k}\\ \;=S_{x}\left(\omega \right)H^{⊤}\left(\omega \right) \end{array}$$

And similarly

(91) $$\begin{array}{l} S_{y}\left(\omega \right)=\sum_{k=-\infty }^{\infty }\langle {\hat{y}}_{m}{\hat{y}}_{m+k}^{⊤}\rangle e^{-i\omega k}\\ \;=H\left(-\omega \right)S_{x}\left(\omega \right)H^{⊤}\left(\omega \right) \end{array}$$

where in the second equality here we used an almost identical derivation as for *S*_**xy**_(ω).

Note that if instead

$$y\left(\omega \right)=H_{x}\left(\omega \right)x\left(\omega \right)+H_{z}\left(\omega \right)z\left(\omega \right),$$

where **x** and **z** are two uncorrelated signals, then we can write

y(ω)=H(ω)v(ω),

where

$$H\left(\omega \right)=\left[\begin{array}{cc} H_{x}\left(\omega \right) & 0\\ 0 & H_{z}\left(\omega \right) \end{array}\right],\;v\left(\omega \right)=\left[x\left(\omega \right),z\left(\omega \right)\right].$$

Thus Equations (90) and (91), respectively give

(92) $$S_{xy}\left(\omega \right)=S_{x}\left(\omega \right)H_{x}^{⊤}\left(\omega \right),\;$$

(93) $$S_{y}\left(\omega \right)=H_{x}\left(-\omega \right)S_{x}\left(\omega \right)H_{x}^{⊤}\left(\omega \right)+H_{z}\left(-\omega \right)S_{z}\left(\omega \right)H_{z}^{T}\left(\omega \right)\text{​}.\;$$

#### 4.4.2. The second order statistics of our system

Previously, we derived Equations (11, 12), which describe the neuronal dynamics using a linear system, written in “state-space” form

(94) $${\hat{s}}_{m+1}=F{\hat{s}}_{m}+d{\hat{T}}_{m}+a{\hat{Y}}_{m}+n_{m},$$

(95) $${\hat{Y}}_{m}=w^{⊤}{\hat{s}}_{m}+e_{m}$$

where **n**_*m*_, *e*_*m*_ and ${\hat{T}}_{m}$ are uncorrelated, zero mean processes with the PSDs Σ_**n**_ ≜ T_*_D (p_*_, T_*_, s_*_), σ^2^_*e*_ = p_*_ (1 − p_*_) and *S*_*T*_(ω), respectively.

In order to apply Equations (92) and (93) to our system we first need to find the transfer function of the system. Applying the Fourier transform to Equations (94, 95) gives

(96) $$e^{i\omega }\hat{s}\left(\omega \right)=F\hat{s}\left(\omega \right)+d\hat{T}\left(\omega \right)+a\hat{Y}\left(\omega \right)+n\left(\omega \right),$$

(97) $$\hat{Y}\left(\omega \right)=w^{⊤}\hat{s}\left(\omega \right)+e\left(\omega \right).$$

Re-arranging terms, we obtain

(98) $$\hat{s}\left(\omega \right)=H_{c}\left(\omega \right)\left(n\left(\omega \right)+d\hat{T}\left(\omega \right)+ae\left(\omega \right)\right),$$

(99) $$\hat{Y}\left(\omega \right)=w^{⊤}H_{c}\left(\omega \right)\text{​}\left(n\text{​}\left(\omega \right)+d\hat{T}\left(\omega \right)+ae\left(\omega \right)\right)+e\left(\omega \right),$$

where we denoted

$$H_{c}\left(\omega \right)≜{\left(Ie^{i\omega }-F-aw^{⊤}\right)}^{-1}.$$

This gives the “closed loop” transfer functions of the system (including the effect of the feedback $\hat{Y}(\omega )$). Next, combining Equations (98, 99) and Equations (92, 93) leads to explicit expressions for the PSDs and CPSDs.

(100) $$S_{sT}\left(\omega \right)=H_{c}\left(-\omega \right)dS_{T}\left(\omega \right)$$

(101) $$S_{s}\left(\omega \right)=H_{c}\left(-\omega \right)\text{​}\left(\Sigma_{n}+aa^{⊤}\sigma_{e}^{2}+dd^{⊤}S_{T}\text{​}\left(\omega \right)\text{​}\right)\text{​}H_{c}^{⊤}\text{​}\left(\omega \right)\text{​},\;$$

(102) $$S_{YT}\left(\omega \right)=w^{⊤}H_{c}\left(-\omega \right)dS_{T}\left(\omega \right),$$

(103) $$\begin{array}{l} \text{​​​}S_{Y}\left(\omega \right)=w^{⊤}H_{c}\left(-\omega \right)\left(\Sigma_{n}+dd^{⊤}S_{T}\left(\omega \right)\right)H_{c}^{⊤}\left(\omega \right)w\\ \;+\sigma_{e}^{2}{\left|1+w^{⊤}H_{c}\left(-\omega \right)a\right|}^{2}. \end{array}$$

For low frequencies it is sometimes more convenient to use the “continuous-time” versions of the PSDs, *S*_**xy**_(*f*) ≜ T_*_S_**xy**_(ω)_ω = 2π fT_*__ for *f* ≪ *T*^−1^_*_, which are approximated by

$$S_{sT}\left(f\right)=T_{*}^{-1}H_{c}\left(-f\right)dS_{T}\left(f\right)$$

(104) $$\begin{array}{l} S_{s}\left(f\right)=H_{c}\left(-f\right)(D\left(p_{*},T_{*},s_{*}\right)+T_{*}^{-1}aa^{⊤}\sigma_{e}^{2}+T_{*}^{-2}dd^{⊤}\\ \;S_{T}\left(f\right))H_{c}^{⊤}\left(f\right), \end{array}$$

(105) $$S_{YT}\left(f\right)=T_{*}^{-1}w^{⊤}H_{c}\left(-f\right)dS_{T}\left(f\right),$$

(106) $$\begin{array}{l} S_{Y}\left(f\right)=w^{⊤}H_{c}\left(-f\right)\left(D\left(p_{*},T_{*},s_{*}\right)+T_{*}^{-2}dd^{⊤}S_{T}\left(f\right)\right)\\ \;H_{c}^{⊤}\left(f\right)w\\ \;+T_{*}\sigma_{e}^{2}{\left|1+T_{*}^{-1}w^{⊤}H_{c}\left(-f\right)a\right|}^{2}. \end{array}$$

where

$$H_{c}\left(f\right)={\left(2\pi fiI-A\left(p_{*},T_{*}\right)-T_{*}^{-1}aw^{⊤}\right)}^{-1},$$

and we used the fact that **F** = **I** + T_*_A (p_*_, T_*_) (Equation 72) and **Σ**_**n**_ = T_*_D (p_*_, T_*_, s_*_) (Equation 77).

Note that if the dimension of **s** is finite and there is no degeneracy, we can always write

(107) $$S_{Y}\left(f\right)=c_{0}+\sum_{j=1}^{M}\frac{c_{j}}{{\left(2\pi f\right)}^{2}+\lambda_{j}^{2}},$$

where λ_*i*_, the poles of *S*_*Y*_(*f*), are determined solely by the poles of **H**_*c*_(*f*) and *S*_*T*_(*f*), while all the other parameters in Equation (106) affect only the constants *c*_*j*_. Commonly, *S*_*T*_(*f*) has no poles – for example, if *S*_*T*_(*f*) is constant so *T*_*m*_ is a renewal process (e.g., the stimulation is periodic or Poisson). Therefore all poles of *S*_*Y*_(*f*) (or the other PSDs) are determined by **H**_*c*_(*f*), i.e., λ_*j*_ are the roots of the characteristic polynomial

(108) $$\left|\lambda I-A\left(p_{*},T_{*}\right)-T_{*}^{-1}aw^{⊤}\right|=0.$$

#### 4.4.3. Spectral factorization

Equations (96) and (97) can be re-arranged as a *direct* I/O relation, formulated, for convenience, in the frequency domain (this can be either *f* or ω – in the section we use ω for brevity of notation, and *f* in other places). Specifically, this relation is of the form

(109) $$\hat{Y}\left(\omega \right)=H^{\text{ext}}\left(\omega \right)\hat{T}\left(\omega \right)+H^{\text{int}}\left(\omega \right)v\left(\omega \right),$$

so *v*_*m*_ = 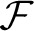
^−1^ (*v*(ω)) is a single **scalar** “noise” process with zero mean and PSD σ^2^_*v*_ (here 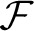
^−1^ is the inverse Fourier transform). This *v*_*m*_ process combines the contributions of *e*_*m*_ and **n**_*m*_, which are the noise processes in the original system (in Equations 96, 97). Such a description, as in Equation (109), describes concisely the contributions of the input and noise to the output (an ARMAx model Lejung, 1999). Using 92 and 93 we respectively find that

(110) $$S_{YT}\left(\omega \right)=H^{\text{ext}}\left(-\omega \right)S_{T}\left(\omega \right)$$

(111) $$S_{Y}\left(\omega \right)={\left|H^{\text{ext}}\left(\omega \right)\right|}^{2}S_{T}\left(\omega \right)+{\left|H^{\text{int}}\left(\omega \right)\right|}^{2}\sigma_{v}^{2}.$$

Comparing Equation (102) with (110) we obtain

(112) $$H^{\text{ext}}\left(\omega \right)=w^{⊤}H_{c}\left(\omega \right)d.$$

Comparing Equation (103) with (111), while using Equation (112), will yield the equation

(113) $$\begin{array}{l} {\left|H^{\text{int}}\left(\omega \right)\right|}^{2}\sigma_{v}^{2}=w^{⊤}H_{c}\left(-\omega \right)\Sigma_{n}H_{c}^{⊤}\left(\omega \right)w+\sigma_{e}^{2}|1\\ \;{+w^{⊤}H_{c}\left(-\omega \right)a|}^{2}. \end{array}$$

This is a “spectral factorization” problem (Anderson and Moore, 1979), with solution

(114) $$H^{\text{int}}\left(\omega \right)=w^{⊤}H_{c}\left(\omega \right)K+1,$$

where

(115) $$K=a+FPw\sigma_{v}^{-2}$$

and

(116) $$\sigma_{v}^{2}=w^{⊤}Pw+\sigma_{e}^{2},$$

with **P** the solution of

(117) $$P=FPF^{⊤}-{\left(w^{⊤}Pw+\sigma_{e}^{2}\right)}^{-1}FPww^{⊤}PF^{⊤}+\Sigma_{n},$$

derived from the general discrete-time algebraic Riccati equation. This can be verified by substitution

$$\begin{array}{l} \;w^{⊤}H_{c}\left(-\omega \right)\Sigma_{n}H_{c}^{⊤}\left(\omega \right)w+\sigma_{e}^{2}{\left|1+w^{⊤}H_{c}\left(\omega \right)a\right|}^{2}\\ \;-{\left|H^{\text{int}}\left(\omega \right)\right|}^{2}\sigma_{v}^{2}\\ =w^{⊤}H_{c}\left(-\omega \right)\left(P-FPF^{⊤}+\sigma_{v}^{-2}FPww^{⊤}PF^{⊤}\right)H_{c}^{⊤}\left(\omega \right)w\\ \;+\sigma_{e}^{2}{\left|1+w^{⊤}H_{c}\left(\omega \right)a\right|}^{2}\\ -{\left|w^{⊤}H_{c}\left(\omega \right)\left(a+FPw\sigma_{v}^{-2}\right)+1\right|}^{2}\sigma_{v}^{2}\\ \overset{\left(1\right)}{=}\text{​}[w^{⊤}H_{o}\left(-\omega \right)\text{​}\left(P-FPF^{⊤}+\sigma_{v}^{-2}FPww^{⊤}PF^{⊤}\right)H_{o}^{⊤}\left(\omega \right)w+\sigma_{e}^{2}\\ -{\left|w^{⊤}H_{o}\left(\omega \right)FPw\sigma_{v}^{-2}+1\right|}^{2}\sigma_{v}^{2}]{\left|1-w^{⊤}H_{o}\left(\omega \right)a\right|}^{-2}\\ =[w^{⊤}H_{o}\left(-\omega \right)\left(P-FPF^{⊤}\right)H_{o}^{⊤}\left(\omega \right)w-w^{⊤}Pw\\ -w^{⊤}H_{o}\left(\omega \right)FPw-w^{⊤}H_{o}\left(-\omega \right)FPw]{\left|1-w^{⊤}H_{o}\left(\omega \right)a\right|}^{-2}\\ \overset{\left(2\right)}{=}[w^{⊤}\left(FH_{o}\left(\omega \right)+I\right)P\left(F^{⊤}H_{o}^{⊤}\left(\omega \right)+I\right)w+w^{⊤}H_{o}\left(-\omega \right)FPF^{⊤}\\ \;H_{o}^{⊤}\left(\omega \right)w-w^{⊤}Pw\\ -w^{⊤}H_{o}\left(\omega \right)FPw-w^{⊤}H_{o}\left(-\omega \right)FPw]{\left|1-w^{⊤}H_{o}\left(\omega \right)a\right|}^{-2}\\ \text{= 0} \end{array}$$

where in (1) we used the fact that **w**^⊤^ **H**_*c*_(ω) = **w**^⊤^ **H**_*o*_(ω) (1 − **w**^⊤^ **H**_*o*_(ω) **a**)^−1^ from the Sherman–Morrison lemma, with **H**_*o*_(ω) = (*e*^*i*ω^ **I** − **F**)^−1^ being the “open loop” version of **H**_*c*_(ω) (i.e., if **a** was zero), and in (2) we used the fact that **H**_*o*_(ω) = *e*^−*i*ω^(**FH**_*o*_(ω) + **I**).

#### 4.4.4. Optimal linear estimation of linear systems

Given that the neuronal dynamics are given by the linear system in Equations (96, 97), there are two different estimation problems one may be interested in. We may want to estimate, based on the history of the previous inputs and outputs ${\left\{{\hat{T}}_{k},{\hat{Y}}_{k}\right\}}_{k=-\infty }^{m\;-\;1}$, either the *parameters* of the model (**F, w, a, d**, σ_*e*_ and Σ_**n**_), or the *variables* in the model (${\hat{Y}}_{m}$ or ${\hat{s}}_{m}$). The first problem is generally termed a “system identification” problem (Lejung, 1999), while the second is a “filtering” (or prediction) problem (Anderson and Moore, 1979). Both are intimately related, and sometimes the solution of the second problem can yield a method of solving the first problem (e.g., section 3.3 in Anderson and Moore, 1979).

A relatively simple way to approach the second (filtering) problem involves the output decomposition we have found in section 4.4.3

$$\hat{Y}\left(\omega \right)=w^{⊤}H_{c}\left(\omega \right)d\hat{T}\left(\omega \right)+\left(w^{⊤}H_{c}\left(\omega \right)K+1\right)v\left(\omega \right).$$

Using this decomposition we can now write a new state-space representation for the system in terms of new state variable ${\hat{z}}_{m}$,

$$\begin{array}{l} {\hat{z}}_{m+1}=\left(F+aw^{⊤}\right){\hat{z}}_{m}+d{\hat{T}}_{m}+Kv_{m},\\ \;{\hat{Y}}_{m}=w^{⊤}{\hat{z}}_{m}+v_{m}, \end{array}$$

which has the same output in the frequency domain (recall, from linear systems theory, that a single I/O relation can be generated by multiple state space realizations). This “innovation form” is particularly useful, since, given the entire history of the previous inputs and outputs $H_{m\;-\;1}≜{\left\{{\hat{T}}_{k},{\hat{Y}}_{k}\right\}}_{k=-\infty }^{m\;-\;1}$, we can recursively estimate the current state precisely (with zero error) (Anderson and Moore, 1979)

(118) $${\hat{z}}_{m}=\left(F+aw^{⊤}\right){\hat{z}}_{m-1}+d{\hat{T}}_{m-1}+K\left({\hat{Y}}_{m-1}-w^{⊤}{\hat{z}}_{m-1}\right).$$

Given this precise estimate of ${\hat{z}}_{m}$, the best linear estimate of ${\hat{Y}}_{m}$ is simply

$$\langle {\hat{Y}}_{m}|H_{m-1}\rangle =w^{⊤}{\hat{z}}_{m}$$

and the estimation error is simply

$$\langle {\left({\hat{Y}}_{m}-\langle {\hat{Y}}_{m}|H_{m-1}\rangle \right)}^{2}\rangle =\langle v_{m}^{2}\rangle =\sigma_{v}^{2}.$$

Since both the innovation form and the original form have the same second order statistics for the input–output, the optimal linear estimator (and its error) for ${\hat{Y}}_{m}$ in the original system would be the same. Moreover, one can show (Anderson and Moore, 1979) that Equation (118) will also give the optimal linear estimate of ${\hat{s}}_{m}$ in the original system, and with error **P** (Equation 117). This solution is the well-known “Kalman filter.”

#### 4.4.5. Example – HHS model power spectral densities

Substituting the parameters for the linearized map (Equations 85–89) into the expressions for the power-spectral densities (Equations 104–106), gives

(119) $$S_{Y}\text{​}\left(f\right)\text{​}=\text{​}\frac{w^{2}\text{​}\left(D_{*}+T_{*}^{-2}d^{2}S_{T}\text{​}\left(f\right)\text{​}\right)\text{​}+\text{​}T_{*}\sigma_{e}^{2}\text{​}\left(\text{​}{\left(2\pi f\right)}^{2}+A_{*}^{2}\right)}{{\left(2\pi f\right)}^{2}+{\left(A_{*}+T_{*}^{-1}wa\right)}^{2}}$$

(120) $$S_{s}\left(f\right)=\frac{D_{*}+T_{*}^{-1}a^{2}\sigma_{e}^{2}+T_{*}^{-2}d^{2}S_{T}\left(f\right)}{{\left(2\pi f\right)}^{2}+{\left(A_{*}+T_{*}^{-1}wa\right)}^{2}}$$

(121) $$S_{YT}\left(f\right)=\frac{T_{*}^{-1}wd}{2\pi fi-A_{*}-T_{*}^{-1}wa}S_{T}\left(f\right).$$

Note that when *S*_*T*_(*f*) ≡ 0 (i.e., periodical spike stimulus), *S*_*Y*_(*f*) has the shape of high pass filter (Figure 3B, *top*). In contrast, *S*_*s*_(*f*) (Figure 3B, *bottom*) and *S*_*YT*_(*f*) both have the shape of a low pass filter (Figure 3D, *top*). From Equations (111) and (110) we know that *S*_*Y*_(*f*) = |*H*^int^(*f*)|^2^σ^2^_*v*_ and *S*_*YT*_(*f*) = *H*^ext^(*f*)*S*_*T*_(*f*), respectively. Therefore, this indicates that *H*^int^(*f*) and *H*^ext^(*f*) are high pass and low pass filters, respectively.

#### 4.4.6. Power spectral densities of response features

So far we have concentrated on the PSD of the response *Y*_*m*_. However, it is easy to extend our formalism to derive the PSDs of different features of the AP, such as its latency or amplitude. We exemplify this on the latency. In Soudry and Meir (2012b) we showed (Figure 3) that for deterministic CBMs, the latency of the AP generated in response to the *m*-th stimulation can be written as a function of the excitability *L*_*m*_ = *L* (**s**_*m*_). In a stochastic model, we have instead

$$L_{m}=\{\begin{array}{l} \begin{array}{l} L\left(s_{m}\right)+\phi_{m}\;,Y_{m}=1\\ \text{not defined },Y_{m}=0 \end{array} \end{array}$$

where ϕ_*m*_ is a zero mean, white noise process generated by the stochasticity of the rapid system. Since it is problematic to define the PSD of *L*_*m*_ if sometimes *Y*_*m*_ = 0, we focus on the case that p_*_ = 1, so we always have *Y*_*m*_ = 1. In this case, assuming again that the perturbations in ${\hat{s}}_{m}$ are small, we can linearize

$$L\left(s_{m}\right)\approx L\left(s_{*}\right)+l^{⊤}{\hat{s}}_{m}$$

where **l** = ∇*L* (**s**)_**s** = s_*__, to obtain (using Equation 11)

(122) $${\hat{s}}_{m+1}=F{\hat{s}}_{m}+d{\hat{T}}_{m}+n_{m},$$

(123) $${\hat{L}}_{m}=l^{⊤}{\hat{s}}_{m}+\phi_{m}$$

where he **F** = **I** + T_*_A (1, T_*_). Therefore, it is straightforward to show that the PSD of the latency is

(124) $$S_{L}\left(f\right)=l^{⊤}S_{s}\left(f\right)l+T_{*}\sigma_{\phi }^{2}$$

where σ^2^_ϕ_ = 〈ϕ^2^_*m*_〉. Note that if latency is a good indicator of excitability, i.e., ***L* (**s**)** changes similarly to *p* (**s**) so that **l** ∝ **w**, then *S*_*L*_(*f*) = *c*_1_*S*_*Y*_(*f*) + *c*_2_ for some constants *c*_1_, *c*_2_, when the input is periodic (*T*_*m*_ = T_*_) and p_*_ → 1.

### 4.5. Numerical tests

MATLAB (2010b) code is available on the ModelDB website, with accession number 144993. In all the numerical simulations of the full stochastic Biophysical neuron model we used Equations (1–3) in main text. We used first order Euler–Maruyama integration with a time step of *dt* = 5 μs (quantitative results were verified also at *dt* = 0.5 μs). Each stimulation pulse was given as a square pulse with a width of *t*_stim_ = 0.5 ms and amplitude *I*_0_ (which were respectively named *t*_0_ and *I*_0_ in Soudry and Meir, 2012b). The results are not affected qualitatively by our choice of a square pulse shape. We define an AP to have occurred if, after the stimulation pulse was given, the measured voltage has crossed some threshold *V*_th_ (we use *V*_th_ = −10 mV in all cases). In all cases where direct stimulation is given, unless stated otherwise, we used periodic stimulation with *I*_0_ = 7.9 μA and T_*_ = 50 ms. Note that for the parameter values used, no APs are spontaneously generated, consistently with experimental results (Gal et al., 2010).

**Figure 8:**
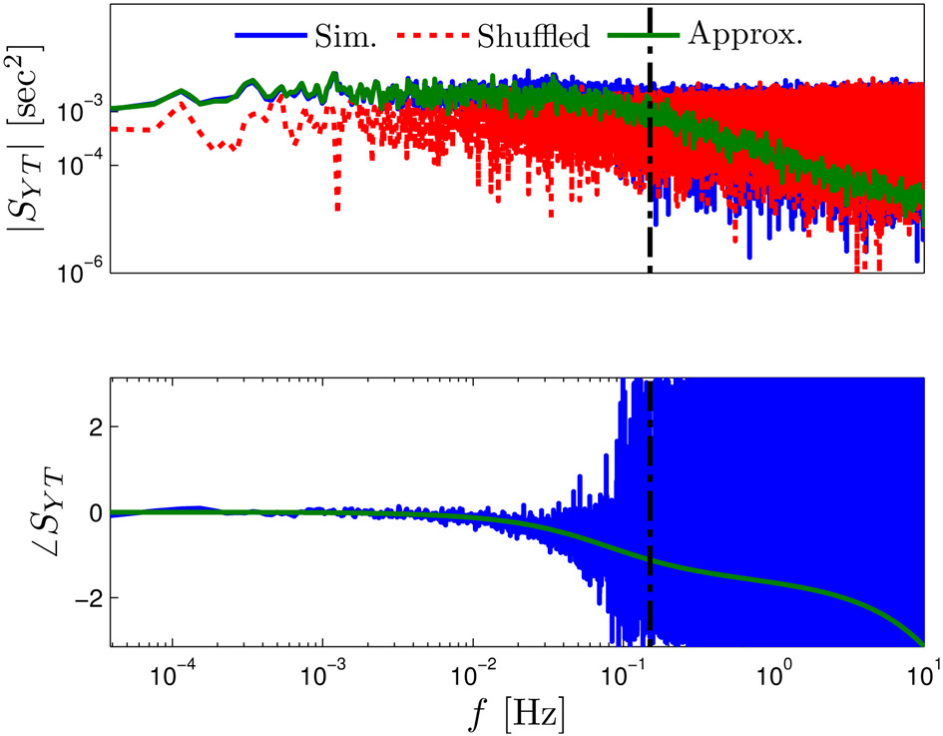
**Estimation noise in the cross-power spectral density**. To estimate the level of this noise in Figure 3D, we added $S_{Y\tilde{T}}\left(f\right)$ where $\left\{{\tilde{T}}_{m}\right\}$ is a shuffled version of {*T*_*m*_}. Only when the estimated *S*_*YT*_(*f*) is above $S_{Y\tilde{T}}\left(f\right)$, is its estimation valid. Therefore, in Figure 3D we show only this region (left of dashed black line), where estimation is valid.

The PSDs were estimated using the Welch method and averaged over eight windows, unless 1/f behavior was observed, in which case we used a single window instead, since long term correlations may generate bias if averaging is used (Beran, 1994). Numerical estimation of the cross-PSD is more problematic. When estimating cross-spectra, estimation noise level can be quite high (proportional to the inverse coherence, according to Bendat and Piersol, 2000, p. 321). To estimate the level of estimation noise, we estimate the cross-spectrum with the input randomly shuffled (Figure 8). Since in this case there is no input–output correlation, this new estimate is pure noise. Finally, as suggested by the reviewer, we smoothed the resulting PSD (or cross-PSD) in all figures (except in Figure 8, where we aimed to show the level of estimation noise). To achieve uniform variance with low bias, we divided the spectrum into 30 logarithmically spaced segments from *f* = 10^−3^ Hz to the maximal frequency (T_*_/2). In each segment *n* the PSD (or cross-PSD) was smoothed using a window of size *n*.

Next, we describe the models used Figures 3–5 and provide their parameter values. These models have either been studied in the literature or are extensions of such models, which are meant to explore the limit for the validity of our analytic approximations. In all cases where direct stimulation is given, unless stated otherwise, we use periodic stimulation with *I*_0_ = 7.9 μA and T_*_ = 50 ms. Notice the form of the models is given in the (more popular) compressed formalism (section 4.1.1), which employs the normalization of state occupation probability to reduce the dimensionality of equations of Equations (2, 3) in the main text.

#### 4.5.1. The HHS model

The HHS model combines the Hodgkin–Huxley equations (Hodgkin and Huxley, 1952) with slow sodium inactivation (Chandler and Meves, 1970; Fleidervish et al., 1996). The model equations (Soudry and Meir, 2012b), which employ the uncoupled stochastic noise approximation, are

$$\begin{array}{l} C\dot{V}={\bar{g}}_{Na}sm^{3}h\left(E_{Na}-V\right)+{\bar{g}}_{K}n^{4}\left(E_{K}-V\right)\\ \;+{\bar{g}}_{L}\left(E_{L}-V\right)+I\left(t\right)\\ \;\dot{m}=\phi \left[\alpha_{m}\left(V\right)\left(1-m\right)-\beta_{m}\left(V\right)m\right]\\ \;+\sqrt{N_{m}^{-1}\phi \left(\alpha_{m}\left(V\right)\left(1-m\right)+\beta_{m}\left(V\right)m\right)}\xi_{m}\\ \;\dot{n}=\phi \left[\alpha_{n}\left(V\right)\left(1-n\right)-\beta_{n}\left(V\right)n\right]\\ \;+\sqrt{N_{m}^{-1}\phi \left(\alpha_{n}\left(V\right)\left(1-n\right)+\beta_{h}\left(V\right)n\right)}\xi_{n}\\ \;\dot{h}=\phi \left[\alpha_{h}\left(V\right)\left(1-h\right)-\beta_{h}\left(V\right)h\right]\\ \;+\sqrt{N_{h}^{-1}\phi \left(\alpha_{h}\left(V\right)\left(1-h\right)+\beta_{h}\left(V\right)h\right)}\xi_{h}\\ \;\dot{s}=\delta \text{​}\left(V\right)\text{​}\left(1-s\right)-\gamma \text{​}\left(V\right)s+\sqrt{N_{s}^{-1}\text{​}\left(\delta \left(V\right)\left(1-s\right)+\gamma \left(V\right)s\right)}\xi_{s}. \end{array}$$

Most of the parameters are given their original values (as in Hodgkin and Huxley, 1952; Fleidervish et al., 1996):

$$\begin{array}{l} \;V_{Na}=50\text{mV},\;V_{K}=-77\text{mV},\;V_{L}=-54\text{mV},\\ {\bar{g}}_{Na}=120{\left(k\Omega \cdot cm^{2}\right)}^{-1},{\bar{g}}_{K}=36{\left(k\Omega \cdot cm^{2}\right)}^{-1},g_{L}=0.3{\left(k\Omega \cdot cm^{2}\right)}^{-1},\\ \;\alpha_{n}(V)=\frac{0.01(V+55)}{1-e^{-0.1\cdot \left(V+55\right)}}\text{kHz},\;\beta_{n}(V)=0.125\cdot e^{-(V+65)/80}\text{kHz},\\ \;\alpha_{m}(V)=\frac{0.1(V+40)}{1-e^{-0.1\cdot (V+40)}}\text{kHz},\;\beta_{m}(V)=4\cdot e^{-(V+65)/18}\text{kHz},\\ \alpha_{h}(V)=0.07\cdot e^{-(V+65)/20}\text{kHz},\;\beta_{h}(V)={\left(e^{-0.1\cdot (V+35)}+1\right)}^{-1}\text{kHz}, \end{array}$$

where in all the rate functions *V* is used in units of mV. In order to obtain the specific spike shape and the latency transients observed in cortical neurons, some of the parameters were modified to

(125) $$\begin{array}{l} \;C_{m}=0.5\;\mu \text{F}/{\text{cm}}^{2},\phi =2\\ \gamma \text{​}\left(V\right)=0.51\cdot \text{​}{\left(e^{-0.3\cdot (V+17)}+1\right)}^{-1}\text{Hz},\delta \text{​}\left(V\right)=0.05e^{-\left(V+85\right)/30}\text{Hz}.\; \end{array}$$

We emphasize that these specific choices do not affect any of our general arguments, but were chosen for consistency with experimental results (Gal et al., 2010). Estimates of channel number vary greatly (Soudry and Meir, 2012b). For simplicity, we chose *N* = *N*_*n*_ = *N*_*h*_ = *N*_*m*_ = *N*_*s*_, and unless stated otherwise, we chose, by default *N* = 10^6^, as in Soudry and Meir (2012b). Note that the HHS model is the same model presented in the paper with *M* = 1, ϕ_*s*,1_ = 1, *N*_*s*,1_ = *N, N*_*r, j*_ = *N* and ϕ_*r*_ = ϕ.

#### 4.5.2. The coupled HHS model

The coupled version of the HHS model uses the same parameters as the uncoupled version, and a similar voltage equation

$$\begin{array}{l} C\dot{V}={\bar{g}}_{Na}s_{0}m_{0}h_{0}\left(E_{Na}-V\right)+{\bar{g}}_{K}n_{0}\left(E_{K}-V\right)\\ \;+{\bar{g}}_{L}\left(E_{L}-V\right)+I\left(t\right) \end{array}$$

where the variables *n*_0_ and *s*_0_*m*_0_*h*_0_ describe the respective fraction of potassium and sodium channels residing in the “open” state. To obtain the coupled model equations, we need to assume something about the structure of the ion channels. The original assumption by Hodgkin and Huxley was that the channel subunits (e.g., *m, n* and *h*) are independent. Over the years, it became apparent that this assumption is inaccurate, and the sodium channel kinetic subunits are, in fact, not independent (Ulbricht, 2005). However, it is not yet clear how the slow sodium inactivation is coupled to the rapid channel kinetics (e.g., Menon et al., 2009; Milescu et al., 2010), so we nevertheless used the original naive HH model assumption that the subunits are independent. In that case the potassium channel structure is given by (for brevity, the voltage dependence on the rates is henceforth ignored for this model)

$$n_{0}\begin{array}{c} \begin{array}{l} 4\alpha_{n}\\ ⇌\\ \beta_{n} \end{array} \end{array}n_{1}\begin{array}{c} \begin{array}{l} 3\alpha_{n}\\ ⇌\\ 2\beta_{n} \end{array} \end{array}n_{2}\begin{array}{c} \begin{array}{l} 2\alpha_{n}\\ ⇌\\ 3\beta_{n} \end{array} \end{array}n_{3}\begin{array}{c} \begin{array}{l} \alpha_{n}\\ ⇌\\ 4\beta_{n} \end{array} \end{array}n_{4}$$

while for the sodium channel it is described by

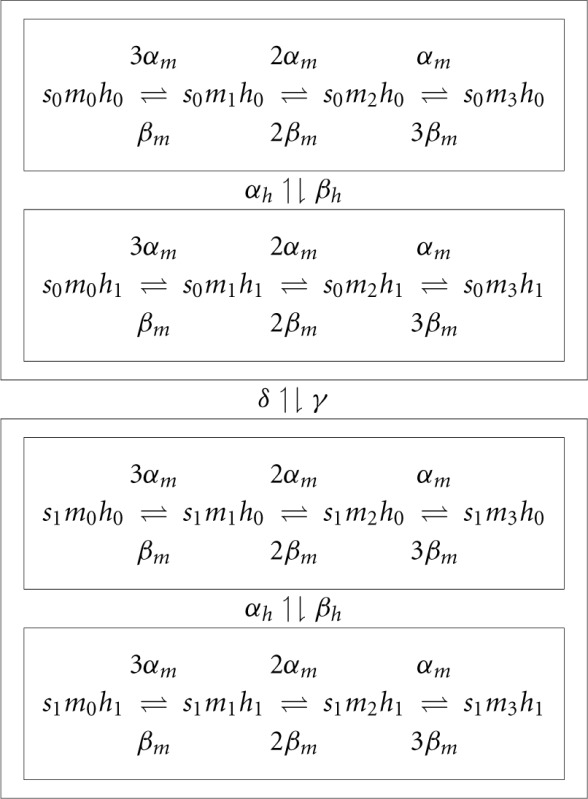



In this diagram, transition rates indicated between two boxed regions, imply that the same rates are used between all corresponding states in boxed regions. The corresponding 32 SDEs are derived using the method described in Orio and Soudry (2012) (or 30 equations if we use the compressed formalism). In this model we used *I*_0_ = 8.3 μA.

#### 4.5.3. The HHSTM model

In order to investigate the effect of a more “physiological” stimulation, we changed the HHS model and added synapses. We used the popular Tsodyks–Markram model for the effect of a synapse with short-term-depression on the somatic voltage (the model first appeared in Tsodyks and Markram (1997) and was slightly corrected in Tsodyks et al. (1998)). In the model *x, y* and *z* are the fractions of resources in the recovered, active and inactive states respectively, interacting through the system

(126) $$\begin{array}{cc} \begin{array}{l} \begin{array}{c} \;y \end{array}\\ \begin{array}{ll} \;↗ & \;↘ \end{array}\\ \begin{array}{lll} x & \leftarrow & z \end{array} \end{array} & . \end{array}$$

Here the *z* → *x* rate is τ^−1^_rec_, the *x* → *y* rate is τ^−1^_in_, and the *x* → *y* rate is *U*_*SE*_δ(*t* − *t*_*sp*_), where δ(·) is the Dirac delta function, and *t*_*sp*_ is the pre-synaptic spike arrival time. The post-synaptic current is given by *I*_*s*_(*t*) = *A*_*SE*_*y*(*t*) where *A*_*SE*_ is a parameter. Additionally, we added noise to the model using the coupled SDE method (Orio and Soudry, 2012), assuming that the diagram in Equation (126), with the corresponding rates, hint at the underlying Markov kinetic structure, with *N* = 10^6^. As in Figure 1B of Tsodyks and Markram (1997), we used τ_in_ = 3 ms, τ_rec_ = 800 ms and *U*_*SE*_ = 0.67. Additionally, we set *A*_*SE*_ = 70 μA to obtain an AP response in our model.

#### 4.5.4. The HHMS model

The HHMS model consists of many sodium currents, each with a different slow kinetic variable. The equations are identical to the HHS model, except that *g*_*Na*_*s* is replaced by *g*_*Na*_*M*^−1^ ∑ ^*M*^_*i*=1_ *s*_*i*_, where *s*_1_ has the same equation as *s* in the HHS model, and for *i* > 2,

$$\begin{array}{l} {\dot{s}}_{i}=\left[\delta \left(V\right)\left(1-s_{i}\right)-\gamma \left(V\right)s_{i}\right]\phi_{s,i}\\ \;+\sqrt{\left(\delta \left(V\right)\left(1-s_{i}\right)+\gamma \left(V\right)s_{i}\right)N_{s,i}^{-1}\phi_{s.i}}\xi_{s,i}, \end{array}$$

with ϕ_*s, i*_ = ϵ^*i*^ and *N*_*s, i*_ = *N*_0_ϵ^*i*η^, where γ (*V*) and δ (*V*) are taken from the HHS model. Unless mentioned otherwise, we chose as default ϵ = 0.2, η = 1.5, *M* = 5, and *N*_0_ = *N* as in Figure 5.

#### 4.5.5. The multiplicative hhms model

The Multiplicative HHMS model is identical to the HHMS model with η = 1, except that ${\bar{g}}_{Na}M^{-1}\sum_{i\;=\;1}^{M}s_{i}$ is replaced with ${\bar{g}}_{Na}\prod_{i\;=\;1}^{M}s_{i}$.

#### 4.5.6. The HHSIP model

The HHSIP model equations (Soudry and Meir, 2012b) are identical to the HHS model equations, except that *s* is renamed to *s*_1_ and an Inactivating Potassium current was added to the voltage equation, where

$$I_{\text{K}}={\bar{g}}_{M}n^{4}s_{2}\left(E_{K}-V\right),$$

with *g*_*M*_ = 0.05*g*_*K*_ and

$$\begin{array}{l} {\dot{s}}_{2}=\delta_{2}\left(V\right)\left(1-s_{2}\right)-\gamma_{2}\left(V\right)s_{2}\\ \;+\sqrt{N_{s_{2}}^{-1}\left(\delta \left(V\right)\left(1-s_{2}\right)+\gamma \left(V\right)s_{2}\right)}\xi_{s,2}, \end{array}$$

where *N*_*s*_2__ = *N* and

$$\begin{array}{l} \delta_{2}\left(V\right)=\frac{3.3e^{(V+35)/15}+e^{-(V+35)/20}}{1+e^{-(V+35)/10}}\text{Hz},\gamma_{2}\left(V\right)\\ \;=\frac{3.3e^{(V+35)/15}+e^{-(V+35)/20}}{1+e^{(V+35)/10}}\text{Hz}. \end{array}$$

Again, in all the rate functions *V* is used in mV units. In this model we used *I*_0_ = 8.3 μA and T_*_ = 33 ms.

#### 4.5.7. The HHMSIP model

The HHMSIP model combines HHSIP and HHMS. Its equations are identical to the HHMS model with η = 2, except they also contain the *I*_*K*_ current from the HHSIP model. In this model we used *I*_0_ = 8.3 μA and T_*_ = 33 ms, unless otherwise specified.

### Conflict of interest statement

The authors declare that the research was conducted in the absence of any commercial or financial relationships that could be construed as a potential conflict of interest.

## Footnotes

^1^A semi-analytic derivation is an analytic derivation in which some terms are obtained by relatively simple numerics. See 2.2 for our implementation.

^2^We demonstrated that such noise should strongly affect the neuronal response to sparse stimulation (Soudry and Meir, 2012b).

^3^I.e., if ∀*t*:*I*(*t*) = 0, then the probability that a neuron will fire is negligible – on any relevant finite time interval (e.g., minutes or days).

^4^E.g., as in Equations (50–52). Note also a similar notation was also used in Soudry and Meir (2012b) (e.g., Equations 2.15, 2.16), where we used *H*/*M*/*L* instead of +/−/0.

^5^Later we shall demonstrate numerically that this is not a necessary condition.

^6^Note a more general Box–Jenkins model is not required, since the poles of *H*^ext^(*f*) and *H*^int^(*f*) are identical (assuming no pole-zero cancelation).

^7^Also, as explained in section 4.3.1, we approximately have *w* ∝ *g*_*Na*_, from Equation (21).
